# Orthogonal monoterpenoid biosynthesis in yeast constructed on an isomeric substrate

**DOI:** 10.1038/s41467-019-11290-x

**Published:** 2019-08-23

**Authors:** Codruta Ignea, Morten H. Raadam, Mohammed S. Motawia, Antonios M. Makris, Claudia E. Vickers, Sotirios C. Kampranis

**Affiliations:** 1 Biochemical Engineering Group, Plant Biochemistry Section, Department of Plant and Environmental Sciences, University of Copenhagen, Thorvaldsensvej 40, 1871 Frederiksberg C, Denmark; 2 Institute of Applied Biosciences – Centre for Research and Technology Hellas (INAB-CERTH), 57001 Thermi, Thessaloniki, Greece; 3 Australian Institute for Bioengineering and Nanotechnology (AIBN), University of Queensland, St. Lucia, QLD 4072 Australia; 4 CSIRO Synthetic Biology Future Science Platform, Commonwealth Scientific and Industrial Research Organisation (CSIRO), GPO Box 2583, Brisbane, QLD 4001 Australia; 5 Present Address: Department of Chemistry, University of Copenhagen, Thorvaldsensvej 40, 1871 Frederiksberg C, Denmark

**Keywords:** Metabolic engineering, Metabolic engineering, Synthetic biology, Metabolic pathways

## Abstract

Synthetic biology efforts for the production of valuable chemicals are frequently hindered by the structure and regulation of the native metabolic pathways of the chassis. This is particularly evident in the case of monoterpenoid production in *Saccharomyces cerevisiae*, where the canonical terpene precursor geranyl diphosphate is tightly coupled to the biosynthesis of isoprenoid compounds essential for yeast viability. Here, we establish a synthetic orthogonal monoterpenoid pathway based on an alternative precursor, neryl diphosphate. We identify structural determinants of isomeric substrate selectivity in monoterpene synthases and engineer five different enzymes to accept the alternative substrate with improved efficiency and specificity. We combine the engineered enzymes with dynamic regulation of metabolic flux to harness the potential of the orthogonal substrate and improve the production of industrially-relevant monoterpenes by several-fold compared to the canonical pathway. This approach highlights the introduction of synthetic metabolism as an effective strategy for high-value compound production.

## Abstract (web summary)

Titers of monoterpenoids production in yeast are low due to the fact that the geranyl diphosphate (GPP)-based pathway can redirect metabolic fluxes to growth. Here, the authors build an orthogonal pathway by selecting the *cis* isomer of GPP as an alternative precursor and achieve high titer monoterpene production.

## Introduction

Synthetic biology approaches for the production of valuable chemicals intervene in the native metabolic pathways of the chassis to balance synthesis of the desired chemical with the production of biomass. However, the structure and regulation of native metabolism has been optimized through evolution to suit the specific needs of the host organism, frequently making it particularly challenging to redirect metabolic fluxes toward the heterologous pathway introduced to synthesize the desirable product. A solution to overcome this obstacle would be to make the heterologous pathway less connected to the chassis’ native metabolism. Ideally, a heterologous pathway must be orthogonal to the host pathways. The concept of orthogonality describes the ability of a system component to be varied without affecting the performance of other components of the same system (e.g., an orthogonal ribosome specifically translates an orthogonal mRNA that is not a substrate for cellular ribosomes)^1^. Although, when used broadly, the term “orthogonal” denotes independence, when applied to biosynthetic pathways, it denotes minimum dependence^2^. This is because the orthogonal pathway still requires precursor influx from the chassis. Thus, for a pathway to be orthogonal, it must have only a single branch point from basic metabolism^3^. Establishing an orthogonal route is advantageous for improved pathway control and performance. The unique branching point can be exploited to create a metabolic valve to redirect fluxes to the orthogonal route and enable dynamic regulation in response to the metabolic status or growth phase^4–9^.

**Fig. 1:**
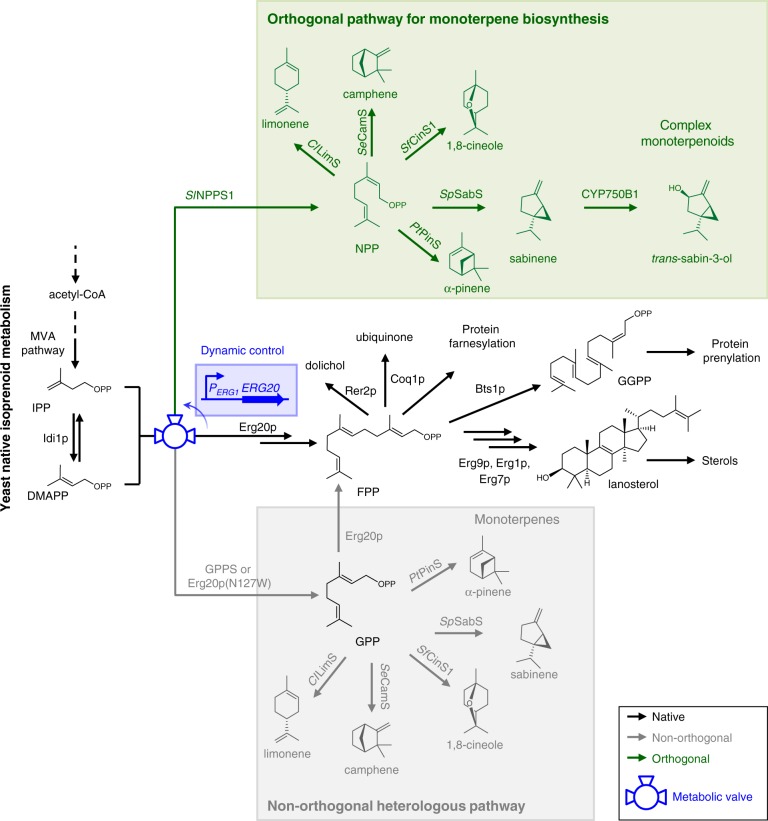
Engineering an orthogonal monoterpene biosynthetic pathway in yeast. The native yeast isoprenoid biosynthesis includes pathways responsible for the synthesis of sterols, dolichols, ubiquinone, as well as protein prenylation. Production of monoterpenoids in yeast has so far been based on utilizing the GPP synthesized as an intermediate in primary isoprenoid metabolism. This GPP-based non-orthogonal pathway (shown in gray) connects back to primary metabolism through the Erg20p-catalyzed conversion of GPP to FPP. Expression of *Sl*NPPS1 in yeast cells enables the synthesis of the *cis*-isomer of GPP, NPP, which cannot be converted to FPP and links back to the biosynthesis of yeast isoprenoids. NPP can be utilized by engineered monoterpene synthases to produce different monoterpenes. The NPP-based pathway, depicted in green, is orthogonal to the yeast metabolism, because it branches out from the main pathway only at one point, following the synthesis of IPP and DMAPP. This branch point creates a metabolic valve, depicted here using the engineering symbol for a three-point valve, which can be regulated to direct fluxes to the desired products. This valve can be controlled dynamically to regulate fluxes between the two branches. We installed dynamic regulation by introducing the ergosterol-repressed promoter of the *ERG1* gene upstream of the *ERG20* gene. This valve diverts fluxes to the orthogonal branch when sterol levels increase. TPS terpene synthase, CYPs cytochrome P450 enzymes, ADHs alcohol dehydrogenases, GGPP geranylgeranyl diphosphate, P_*ERG1*_ ERG1 promoter

A characteristic case, where the establishment of an orthogonal metabolic pathway could be beneficial, is the synthesis of monoterpenoids in *Saccharomyces cerevisiae*. Monoterpenoids are widely used as flavors, antibacterials, and insecticides^10,11^, and recently, their applications have expanded to include high-density fuels, renewable polymers, and green plastics^12^. However, extraction of monoterpenoids from natural sources cannot meet the increasing demand, prompting efforts for their biotechnological production in microorganisms^13–17^. Monoterpene scaffolds are synthesized by the conversion of the canonical 10-carbon terpene precursor geranyl diphosphate (GPP) by monoterpene synthases, which catalyze the rearrangement of the substrate to generate a plethora of structures^18^ (Fig. 1). GPP itself is formed by the fusion of two C_5_ units, dimethylallyl diphosphate (DMAPP) and isopentenyl diphosphate (IPP). Further addition of IPP to GPP generates the 15-carbon farnesyl diphosphate (FPP), a precursor of the sesquiterpenes (C_15_), diterpenes (C_20_), polyprenols, ubiquinone, and sterols.

The yeast *S. cerevisiae* is an attractive host for terpene bioproduction, due to its robustness, applicability to industrial bioprocesses, and the possibility for terpene scaffold decoration by the functional expression of cytochrome P450 enzymes. Metabolic engineering efforts in yeast for the production of FPP-derived compounds, such as the sesquiterpenes artemisinin and farnesene, have achieved industrial-scale levels^19–22^. However, production of GPP-derived compounds has so far been considerably less efficient^14,23,24^. One of the factors contributing to this lower efficiency may be the structure of the isoprenoid biosynthesis pathway in yeast, which is optimized for the production of FPP-derived molecules that are essential for growth and viability. A key feature of the pathway that highlights this metabolic optimization is the sequential nature of FPP synthesis by the native yeast prenyltransferase, Erg20p. Erg20p is a bifunctional enzyme that catalyzes two successive steps of the pathway; initially, it condenses DMAPP and IPP to form GPP, and subsequently it catalyzes the condensation of GPP with one more IPP molecule to generate FPP^25^ (Fig. 1). This facilitates the metabolic economy of the cell^26^, as yeast does not naturally produce other GPP-derived compounds. However, the sequential reaction catalyzed by Erg20p appears to limit the GPP pool, hindering monoterpenoid production^14^. Overexpression of a GPP synthase to increase GPP availability resulted in only a moderate monoterpene increase, because the increased GPP flux was efficiently channeled by the endogenous Erg20p to produce sterols and other essential isoprenoids^14^. Further efforts, in which Erg20p was engineered to be inefficient only in the FPP-synthesizing step, increased the GPP pool without abolishing sterol synthesis^14^. However, despite additional improvements through N-degron-dependent protein degradation of the native Erg20p to minimize competition with the introduced monoterpene synthase^23^, the obtained monoterpene titers were orders of magnitude lower than the titers of sesquiterpene production by other engineered yeast platforms^21,22,24,27^. These observations suggest that there is still considerable potential for improving the monoterpene biosynthesis in yeast.

To overcome limitations in monoterpene production that may be imposed by the structure and regulation of the yeast native metabolism, we set out to establish an orthogonal pathway. To achieve orthogonality, the new pathway must have only one branching point from yeast metabolism. To meet this requirement, the synthetic pathway must not involve GPP because Erg20p connects GPP back to basic yeast metabolism via FPP (Fig. 1). This requirement creates a considerable challenge, as GPP is the precursor of monoterpenes. Thus, we examined the possibility of using alternative precursors for monoterpene biosynthesis. Although it has been shown that GPP is the canonical substrate of monoterpene synthases^18^, recent studies in different tomato species have revealed the existence of a small group of enzymes that deviate from this rule and preferentially convert the *cis*-isomer of GPP, neryl diphosphate (NPP), to a limited number of monoterpene hydrocarbons^28–30^. A dedicated *cis-*prenyltransferase, neryl diphosphate synthase (*Sl*NPPS1), was found to generate the required NPP substrate by condensing DMAPP and IPP in the *cis*-configuration^28^ (Fig. 1). NPP and NPP-derived monoterpene biosynthesis has only been observed in these few plant species and is entirely absent in yeast.

Here, we use NPP as an alternative substrate to establish an orthogonal pathway for monoterpene scaffold production. We identify a single residue dictating isomeric substrate selectivity in a monoterpene synthase and engineer canonical terpene synthases to specifically accept the alternative substrate. As a result, we establish a complete synthetic pathway that extends up to the step of monoterpene oxidation and achieve marked improvements in the production of several industrially important monoterpenes.

## Results

### Justifying the construction of an orthogonal monoterpene pathway

To justify the need for constructing an orthogonal pathway, we first confirmed that the low titers of monoterpene observed in previous efforts^14,23^ were not due to product toxicity, and that additional factors, such as pathway structure, may play an important role. We evaluated the growth of yeast cells engineered to provide high levels of isoprenoid precursors (strain AM94; Supplementary Table 1) under conditions that recapitulate production of specific titers of monoterpenes. In agreement with previous studies^31,32^, yeast cells grew equally well when supplemented with increasing monoterpene concentrations up to 500 mg L^−1^ (Supplementary Fig. 1), which was more than seven times higher than the current best-production titers^23^. Subsequently, we introduced the *Citrus limon* limonene synthase (*Cl*LimS)^33^ into strain AM94 and confirmed that limonene productivity was not reduced, even when the culture was supplemented with a limonene concentration that was tenfold higher than the titer produced by the strain (Supplementary Fig. 2).

To further corroborate previous evidence suggesting that the sequential reaction of Erg20p may be a contributing factor to low monoterpene productivity^14^, we supplemented the cell extract of *Cl*LimS-expressing AM94 cells with isoprenoid precursors and measured the formation of limonene. Addition of GPP, in the presence of IPP and DMAPP, resulted in the production of low amounts of limonene and a significant larger amount of FPP (Supplementary Fig. 3). This suggested that most of the GPP precursor was used by Erg20p to synthesize FPP, and only a small amount of the GPP added was converted to limonene by *Cl*LimS (Supplementary Fig. 3). Further experiments using ^13^C-GPP confirmed that <10% of the ^13^C-GPP was converted to limonene under these conditions, and most of it was channeled to FPP. Therefore, Erg20p is very efficient in utilizing GPP, reducing the GPP pool and hindering monoterpene production. Thus, we set out to engineer an orthogonal pathway that does not depend on GPP.

### Establishing efficient NPP biosynthesis in yeast cells

**Fig. 2:**
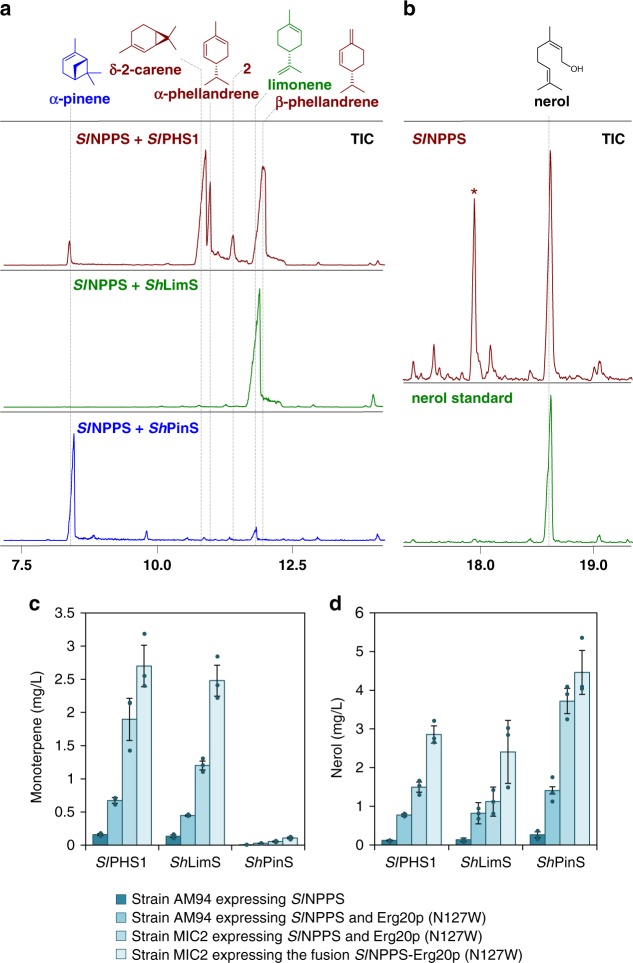
Engineering NPP production in yeast. **a** Three NPP-specific monoterpene synthases were co-expressed in yeast with *Sl*NPPS1. *Sl*PHS1 (brown) produced a mixture of monoterpenes, including β-phellandrene, δ-2-carene, α-phellandrene, and α-terpinene. *Sh*LimS (green) produced exclusively limonene, while *Sh*PinS (blue) synthesized α-pinene as the main product, together with low amounts of limonene. **b** Production of nerol by yeast cells (brown), presumably resulting from the hydrolysis of excess NPP. The peak marked with a star (*) corresponds to a non-terpene yeast-derived compound. The nerol standard is shown in green. **c** Stepwise engineering of NPP production in yeast was monitored by measurement of the monoterpene products synthesized by the three NPP-specific synthases. **d** Production of the nonspecific monoterpene product nerol by the engineered cells increased as monoterpene yields improved, suggesting that the selected NPP-specific synthases could not fully harvest the NPP flux. Error bars correspond to the mean absolute deviation (MAD) around the mean (*n* = 3, corresponding to three separate yeast transformations processed and analyzed independently). Source data of **c** and **d** are provided as a Source Data file

A cDNA fragment encoding *Sl*NPPS1 was cloned into the yeast vector pHTDHmyc under the P_*TDH3*_ constitutive promoter and expressed in strain AM94. To monitor NPP synthesis, we also introduced yeast codon-optimized versions of three different NPP-specific monoterpene synthases, *Solanum lycopersicum* phellandrene synthase (*Sl*PHS1)^28^, *Solanum habrochaites* limonene synthase (*Sh*LimS)^30^, and *S. habrochaites* pinene synthase (*Sh*PinS)^30^. When expressed in yeast in the absence of *Sl*NPPS1, no new products were detected, as none of the three monoterpene synthases were able to utilize the available GPP. However, upon *Sl*NPPS1 co-expression, all three synthases produced their reported NPP-derived products. Specifically, *Sl*PHS1 produced *β*-phellandrene (48.6%), δ-2-carene (36.6%), α-phellandrene (8.3%), and α-terpinene (6.5%). *Sh*LimS produced limonene as a single product, and *Sh*PinS produced α-pinene (84%) and limonene (16%) (Fig. 2a). Product titers in all cases were very low, ranging from 0.009 mg L^−1^ α-pinene to 0.16 mg L^−1^ *β*-phellandrene (Supplementary Table 2). Furthermore, we observed that AM94 cells engineered to express *Sl*NPPS1 in the absence of any monoterpene synthase produced detectable levels of nerol, as confirmed by comparison of mass spectra and retention time with an authentic standard (Fig. 2b). By analogy to the formation of geraniol via the hydrolysis of GPP by intracellular phosphatases like Lpp1p and Dpp1p^14,34^, we attributed the production of nerol to the hydrolysis of NPP that accumulated in the absence of a suitable terpene synthase. Taken together, these results confirmed that the alternative substrate, NPP, is synthesized in the engineered yeast strain and it can be used to produce monoterpenes.

To ensure that NPP can form the basis for an orthogonal pathway, we confirmed that it did not interfere with the rest of the yeast isoprenoid metabolism. With a combination of in vitro and in vivo experiments, we confirmed that NPP was not taken up by Erg20p to produce larger prenyl diphosphates. We expressed Erg20p in *Escherichia coli* and used the purified protein (Supplementary Fig. 4) in in vitro assays with GPP and NPP. Although the recombinant enzyme was able to produce FPP from IPP and GPP, it was not able to catalyze elongation of NPP. We were also unable to detect any NPP-derived products of Erg20p in yeast cells or in yeast cell extracts supplemented with NPP and IPP (Supplementary Fig. 5). We also confirmed that the growth properties of *Sl*NPPS1-expressing yeast cells were very similar to their empty vector-containing counterparts (Supplementary Fig. 6).

### Redirecting flux to the orthogonal pathway

The new branching point established for monoterpene biosynthesis could function as a valve to direct the flux of substrates away from FPP synthesis and to increase the monoterpene yield (Fig. 1). First, we aimed to decrease the competition for IPP by Erg20p. To achieve this, we introduced Erg20p(N127W), a dominant-negative Erg20p variant that inhibits the FPP synthesis step of the endogenous wild-type enzyme^14,35^. This resulted in a 3–4-fold increase (depending on the enzyme) in the total monoterpene yield of all three NPP-specific monoterpene synthases (Fig. 2c and Supplementary Table 2).

Heterozygous gene deletions in yeast have been shown to decrease the level of the corresponding protein to 50%^36^. Thus, to further reduce the flux through the FPP-synthesizing branch, we downregulated Erg20p by shifting to the yeast strain MIC2, which carries a deletion in one of the two *ERG20* alleles^14^. This resulted in a further 1.9–2.8-fold increase in monoterpene titer (Fig. 2c and Supplementary Table 2).

Previous studies had shown that fusion of a heterologous enzyme to Erg20p can be beneficial for the enzyme’s stability^37–39^. We fused *Sl*NPPS1 to the C terminus of Erg20p(N127W) and confirmed that the fused protein reached higher intracellular levels compared with the non-fused *Sl*NPPS1 (Supplementary Fig. 7). We subsequently introduced the Erg20p(N127W)–*Sl*NPPS1 variant into strain MIC2 to achieve an additional 1.4–2-fold improvement in production. The combination of these interventions resulted in an overall 12–18-fold increase, reaching 2.7 mg L^−1^ β-phellandrene, 2.5 mg L^−1^ limonene, and 0.11 mg L^−1^ α-pinene (Fig. 2c and Supplementary Table 2). Following these improvements, a significant amount of nerol could be detected in the NPP-producing cells overexpressing *Sh*LimS, *Sh*PinS, or *Sl*PHS1 (Fig. 2d and Supplementary Table 3). This suggested that the combination of these interventions achieved efficient synthesis of NPP, but utilization of the alternative substrate has reached a bottleneck at the level of the monoterpene synthases that resulted in its accumulation and eventual conversion to nerol.

### Canonical monoterpene synthases accept NPP as a substrate

**Fig. 3:**
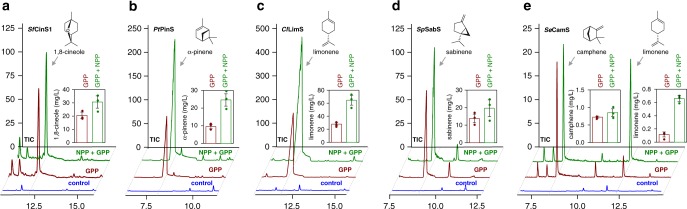
Monoterpene production by canonical terpene synthases. Five different monoterpene synthases were overexpressed in yeast cells producing solely GPP (brown), or also producing NPP as an alternative substrate (green). The monoterpene product titers increased markedly when NPP was also available with all synthases tested: *Salvia fruticosa* 1,8-cineole synthase (*Sf*CinS1) (**a**), *Pinus taeda* α-pinene synthase (*Pt*PinS) (**b**), *Citrus limon* limonene synthase (*Cl*LimS) (**c**), *S. pomifera* sabinene synthase (*Sp*SabS) (**d**), and *S. elaeagnifolium* camphene synthase (*Se*CamS) (**e**). A significant shift from camphene to limonene was observed in the product profile of *Se*CamS with NPP. Yeast cells producing NPP in the absence of any monoterpene synthase are shown as control (blue). Samples were analyzed in triplicate (*n* = 3 biologically independent samples). Error bars for each sample correspond to the mean absolute deviation (MAD) around the mean. Source data of **a**–**e**  are provided as a Source Data file

The accumulation of nerol indicated that the three NPP-specific synthases tested here could not harvest the full potential of the orthogonal pathway, and more efficient biocatalysts were required. However, only few NPP-specific synthases have been reported to date^28,30,40^, limiting the possibility that a search to identify enzymes more active than the synthases used here would be successful. Furthermore, the product range of the known NPP-specific enzymes was limited to only four main products, β-phellandrene^28^, limonene^30^, α-pinene^30^, and nerol^40^. To identify enzymes that are more active and to enable the production of a broad range of different monoterpene scaffolds, we turned into the rich resource of canonical, GPP-converting, plant monoterpene synthases. We selected five synthases with different product profiles, including *Cl*LimS^33^, *Salvia fruticosa* 1,8-cineole synthase (*Sf*CinS1)^41^, *Salvia pomifera* sabinene synthase (*Sp*SabS)^41^, *Pinus taeda α-*pinene synthase (*Pt*PinS)^42^, and *Solanum elaeagnifolium* camphene synthase (*Se*CamS)^43^, and tested whether they can accept NPP as a substrate. When introduced to strain MIC2 expressing Erg20p(N127W), i.e., a GPP-synthesizing strain, all five enzymes produced their expected monoterpene products (Fig. 3). When Erg20p(N127W) was replaced by the Erg20p(N127W)–*Sl*NPPS1 fusion to establish the production of NPP, an overall increase ranging between 1.3- and 2.8-fold (depending on the enzyme) in monoterpene formation compared with the GPP-only producing strain was achieved with all canonical monoterpene synthases (Fig. 3). This suggested that the canonical enzymes could accept both substrates, resulting in overall higher yields, and that the different enzymes had varying capacity to accept the isomeric substrate. We confirmed that the selected monoterpene synthases were able to utilize NPP in in vitro assays with bacterially expressed enzymes (Supplementary Figs. 4 and 8a). We also examined the product profile of the five enzymes with NPP in vitro and found that four of them synthesized almost the same blend of monoterpenes as with GPP. Only the profile of *Se*CamS shifted from camphene toward limonene with NPP in vitro (Fig. 3e; Supplementary Fig. 8a), which was reflected in the product profile of NPP-producing yeast cells expressing *Se*CamS (Supplementary Fig. 8b). There was no new monoterpene product with any of the synthases and NPP.

Kinetic analysis of the reaction with each isomeric substrate revealed that the canonical synthases exhibited higher affinity and overall catalytic efficiency (*k*_cat_^app^/*K*_M_^app^) with GPP than with NPP (*Sf*CinS1: 0.213 min^−1^ μM^−1^ with GPP vs. 0.137 min^−1^ μM^−1^ with NPP; *Cl*LimS: 0.735 min^−1^ μM^−1^ with GPP vs. 0.456 min^−1^ μM^−1^ with NPP; *Se*CamS: 0.21 min^−1^ μM^−1^ with GPP vs. 0.044 min^−1^ μM^−1^ with NPP; Table 1). Yet, the observation that yeast productivity with *Cl*LimS and *Pt*PinS when both the GPP and the NPP pathways were present was more than double, compared with when only the GPP pathway was active (Fig. 3), provided two main conclusions. First, it indicated that these terpene synthases were not the limiting step of the pathway. Second, the ability of *Cl*LimS and *Pt*PinS to support equal or higher titers with NPP, despite this not being their preferred substrate, suggested that the established NPP-based pathway was efficient and had the potential to exceed the GPP pathway in productivity. To further evaluate the performance of the NPP-based pathway, we examined the kinetic characteristics of *Sl*NPPS1 using in vitro assays with enzymes expressed and purified in *E. coli* (Supplementary Fig. 4). The determined kinetic parameters revealed that *Sl*NPPS1 is significantly less efficient than Erg20p in utilizing DMAPP and IPP (Supplementary Table 4; refs. ^14,^^28^). Combined with the preference of canonical terpene synthases for GPP rather than NPP (Table 1), we concluded that the observed increase in productivity is not due to favorable kinetics of the *Sl*NPPS1 branch.

**Table 1:** Kinetic parameters of terpene synthases and variants

Enzyme variant	Substrate	*k*_cat_^app^ (min^−1^)	*K*_M_^app^ (μM)	*k*_cat_^app^/*K*_M_^app^ (min^−1^ μM^−1^)
*Sf*CinS1 wild type	GPP	5.56 ± 0.38	26.12 ± 2.79	0.213
*Sf*CinS1 wild type	NPP	12.2 ± 2.67	89.46 ± 4.99	0.137
*Sf*CinS1(F571Y)	GPP	0.37 ± 0.03	29.93 ± 6.14	0.012
*Sf*CinS1(F571Y)	NPP	0.75 ± 0.12	37.48 ± 14.26	0.020
*Sf*CinS1(F571H)	GPP	0.86 ± 0.09	18.05 ± 5.07	0.048
*Sf*CinS1(F571H)	NPP	0.74 ± 0.08	238.1 ± 34.35	0.003
*Sf*CinS1(F571V)	GPP	0.32 ± 0.08	271.5 ± 95.30	0.001
*Sf*CinS1(F571V)	NPP	0.45 ± 0.04	67.24 ± 13.30	0.007
*Sf*CinS1(F571L)	GPP	0.44 ± 0.07	55.72 ± 9.22	0.008
*Sf*CinS1(F571L)	NPP	0.64 ± 0.08	71.82 ± 17.63	0.009
*Cl*LimS wild type	GPP	18.34 ± 0.88	24.94 ± 3.76	0.735
*Cl*LimS wild type	NPP	37.16 ± 1.76	81.55 ± 7.33	0.456
*Cl*LimS(H570F)	GPP	14.64 ± 2.50	12.86 ± 7.20	1.138
*Cl*LimS(H570F)	NPP	29.70 ± 1.82	31.94 ± 5.14	0.930
*Cl*LimS(H570Y)	GPP	8.92 ± 0.43	11.76 ± 4.82	0.759
*Cl*LimS(H570Y)	NPP	21.86 ± 1.66	23.98 ± 6.41	0.911
*Cl*LimS(H570L)	GPP	0.24 ± 0.07	16.89 ± 6.72	0.014
*Cl*LimS(H570L)	NPP	4.58 ± 0.66	110.5 ± 19.30	0.041
*Cl*LimS(H570V)	GPP	1.16 ± 0.08	56.70 ± 12.42	0.021
*Cl*LimS(H570V)	NPP	1.24 ± 0.10	32.44 ± 14.14	0.038
*Cl*LimS(H570I)	GPP	0.21 ± 0.02	24.43 ± 6.44	0.009
*Cl*LimS(H570I)	NPP	1.90 ± 0.13	27.06 ± 4.72	0.070
*Se*CamS wild type	GPP	3.91 ± 0.22	18.58 ± 2.77	0.210
*Se*CamS wild type	NPP	4.82 ± 0.39	107.49 ± 12.48	0.044

Errors represent standard error of the fitted parameters. Source data are provided in a Source Data file

The canonical monoterpene synthases convert GPP and NPP to the same blend of products (Supplementary Fig. 8), not allowing to determine the relative contribution of the two substrates simply by examining the product profile. To resolve this, we synthesized ^13^C-labeled NPP (Supplementary Fig. 9), so as to distinguish the products of each substrate when mixtures of the labeled compound with unlabeled GPP were used. We added precursors to extracts of yeast cells expressing *Cl*LimS and determined the isotopic composition of the produced limonene using GC–APCI–QqToF analysis. In these experiments, ^13^C-NPP was initially shown to be channeled efficiently to limonene synthesis, while GPP was mostly consumed to produce FPP (Supplementary Figs. 10 and 11). In subsequent competition experiments between ^13^C-labeled NPP and GPP, when the two isomeric substrates were added at equal concentrations, over 90% of the limonene produced originated from ^13^C-NPP. The contribution of the two substrates to limonene production was comparable, only when GPP was in tenfold excess (Supplementary Fig. 12a).

Comparison of the overall production titers revealed that *Cl*LimS and *Pt*PinS were 24 and 224 times more efficient, respectively, in limonene and α-pinene synthesis in NPP-producing yeast cells than their NPP-specific counterparts, *Sh*LimS and *Sh*PinS (Supplementary Table 5). Taken together, these findings confirmed that canonical monoterpene synthases can serve as efficient surrogate synthases in this synthetic pathway and suggested that canonical enzymes could offer a starting point for protein engineering efforts to improve NPP utilization.

### Engineering NPP-specific monoterpene synthases

**Fig. 4:**
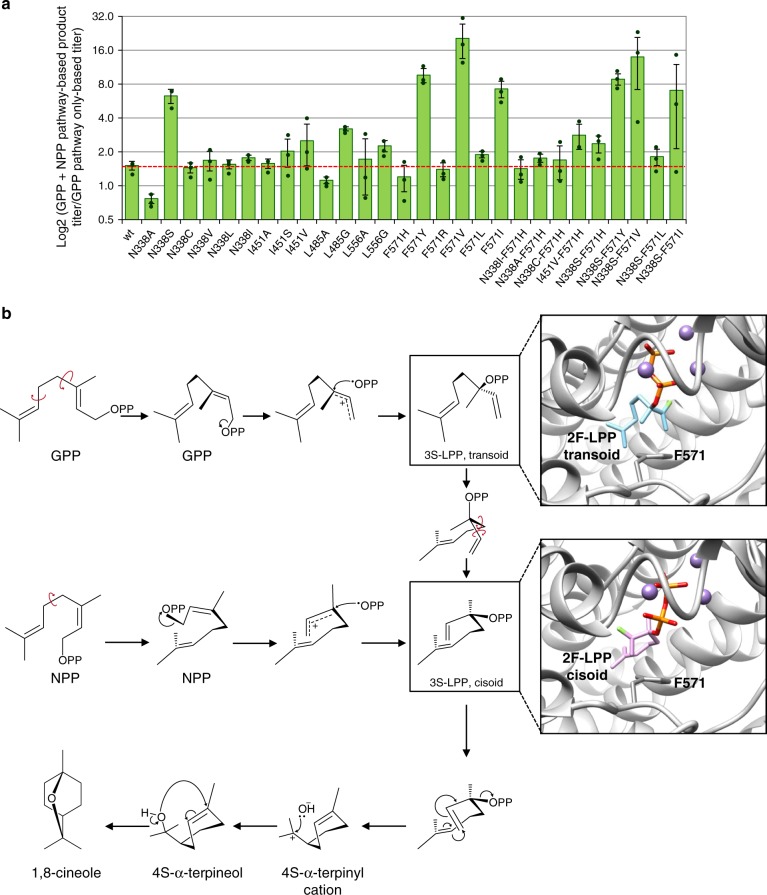
Engineering SfCinS1 to become specific for NPP. **a** A library of 19 different *Sf*CinS variants was evaluated in yeast cells producing either only GPP, or both GPP and NPP. To identify variants with improved specificity for NPP, the ratio of monoterpene product titers obtained with and without the NPP pathway was determined and shown here in logarithmic scale. Samples were analyzed in triplicate originating from independent yeast transformations (*n* = 3 biologically independent samples). Errors correspond to the mean absolute deviation (MAD) around the mean. **b** Proposed GPP and NPP cyclization mechanism leading to the synthesis of 1,8-cineole by *Sf*CinS1. Initially, GPP binds at the extended conformation. Following syn migration of the diphosphate, the transoid extended conformation of LPP transitions first to the cisoid form and then to the catalytically competent cisoid closed conformation^44^. In NPP, the C-2,3 bond is already in the *cis-*conformation, bypassing the requirement for the transoid to cisoid transition. The insets show models of the active site of *Sf*CinS1 with two proposed conformers of 2-fluorolinalyl diphosphate (2F-LPP) superimposed. The two conformations have been obtained by soaking crystals of mint limonene synthase with 2F-LPP or 2-fluoro-geranyl diphosphate (2F-GPP). In the crystal, 2F-GPP is converted to the transoid 2F-LPP form, while 2F-LPP assumes a conformation that resembles the extended cisoid form of LPP. For the GPP reaction to proceed, extensive conformational changes of the substrate are needed for the conversion from transoid to cisoid. F571 is positioned in the center of this transition. Red arrows indicate chemical bond rotation. Graphics produced with UCSF Chimera^68^. Source data of **a**  are provided as a Source Data file

To fully exploit the potential of the orthogonal pathway, we set out to engineer the selected canonical monoterpene synthases, with the aim to increase their efficiency and specificity for NPP. Initially, we focused on *Sf*CinS1 due to the availability of structural information and our previous experience in engineering this enzyme to alter its substrate and product specificity^41^. In these efforts, we developed a library of 19 variants at different positions in the active site^39,41^. Here, we differentially screened this library in GPP- and NPP-producing yeast strains to identify sites that potentially influence substrate specificity. As shown in Fig. 4a, one site, F571, stood out as playing a critical role in selecting between the two isomeric substrates, as several variants at this position were able to markedly improve the performance of the enzyme with NPP. Variants *Sf*CinS1(F571V), *Sf*CinS1(F571Y), and *Sf*CinS1(F571I) were 18, 9, and 7 times more efficient, respectively, when the NPP pathway was present, compared with their performance with only GPP as a substrate (Fig. 4a). Although the catalytic efficiency of the variants was lower than that of the wild-type enzyme (Supplementary Table 6), these findings suggested that F571 may be acting as a switch that differentiates substrate preference between NPP and GPP. In addition to F571, certain substitutions in a second residue, N338, also resulted in shifts in specificity, albeit in a less efficient or consistent manner. Variants *Sf*CinS1(N338A) and *Sf*CinS1(N338S) exhibited an opposing substrate selectivity. *Sf*CinS1(N338A) became a strictly GPP-utilizing enzyme, while *Sf*CinS1(N338S) was 6.7 times more efficient when the NPP pathway was present (Fig. 4a). To test for potential synergistic effects, we also constructed several variants combining F571 substitutions with substitutions in the other positions (Supplementary Table 7). However, no further improvements in substrate specificity were observed (Fig. 4a). For further studies, we focused exclusively on the role of F571, because of the extent and consistency of specificity changes.

Analysis of the structural information available for *Sf*CinS1^41^ in the context of biochemical and structural studies on the enzymatic mechanism of monoterpene synthases^44^ provided insight into the possible role of F571. As shown in Fig. 4b, the early steps of the canonical monoterpene synthase mechanism involve the initial binding of GPP in an extended conformation, followed by the ionization of the substrate and the syn migration of the diphosphate moiety to C-3. The resulting linalyl diphosphate (LPP) maintains the transoid conformation of GPP (Fig. 4b). Thus, for cyclization to take place, the C-2,3 bond must rotate to convert LPP from the transoid to the cisoid conformer. Subsequent conformational change of LPP to the closed configuration brings C-6 and C-1 to a position competent for cyclization. Following cyclization, the resulting carbocations rearrange to finally yield different hydrocarbons (Fig. 4b). Due to the cisoid conformation of NPP, its cyclization does not require the same transition from the transoid to the cisoid form (Fig. 4b). As shown in Fig. 4b, in the structure of *Sf*CinS1, F571 is positioned at a short distance from C-1 of LPP. This analysis suggested two possible explanations for the ability of F571 to control isomeric substrate selectivity. Considering the extensive conformational changes that are required for the substrate to transition from the transoid to the cisoid form (compare the structure of the two intermediate analogs in Fig. 4b), one scenario would be that F571 plays a direct role in this process. In this case, substitutions in F571 that impair the transoid to cisoid transition would have a negative impact in the GPP-based reaction, but would not interfere with the transition-independent NPP reaction. Alternatively, F571 may not be directly involved in the transition process; instead, the size and orientation of the side chain at position 571 may alter the geometry of the active site to favor binding of one substrate versus the other. To distinguish between these two mechanisms, we conducted a detailed kinetic analysis of *Sf*CinS1 mutants. *Sf*CinS1(F571V), *Sf*CinS1(F571Y), and *Sf*CinS1(F571L) showed shifts in *K*_M_^app^ either favoring NPP binding or disfavoring binding of GPP (Table 1), suggesting that the residue at position 571 exerts stereochemical control. In agreement, the reverse behavior of *Sf*CinS1(F571H), which showed preference for GPP in vivo, was the result of a significant increase in *K*_M_^app^ for NPP.

**Fig. 5:**
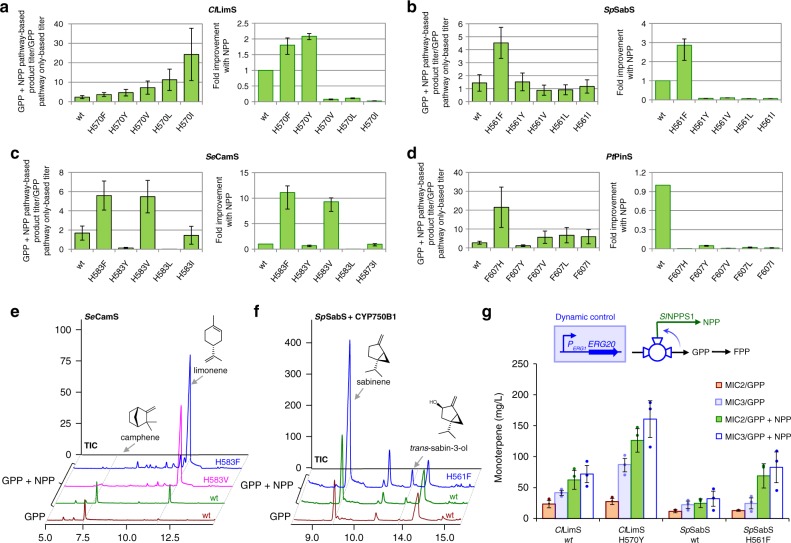
Monoterpene profile of the terpene synthase variants. Variants of *Cl*LimS (**a**), *Sp*SabS (**b**), *Se*CamS (**c**), and *Pt*PinS (**d**), carrying different substitutions in the residue homologous to *Sf*CinS1(F571), were analyzed in yeast cells producing NPP versus non-NPP-producing cells. On the left-hand side of panels **a**–**d**, the bar charts show the ratio of the total monoterpene production titer when both the NPP and GPP pathways are present divided by the titer of monoterpene production by the same variant when only the GPP pathway is present. The bar charts on the right-hand side of panels **a**–**d** show the total monoterpene titer obtained by each mutant synthase when the NPP pathway was present in comparison to the corresponding wild-type enzyme titer (normalized to 1), indicating the overall efficiency of each enzyme variant. **e** *Se*CamS variants H583F and H583V switch specificity from a camphene synthase to a highly efficient limonene synthase. **f** The overall improvements in pathway performance enable the introduction of an additional downstream modification step. Yeast cells co-expressing *Sp*SabS and CYP750B1 produce detectable amounts of *trans*-sabin-3-ol only when the NPP pathway is present. Replacement of wild-type *Sp*SabS by the NPP-specific variant *Sp*SabS(H561F) improved trans-sabin-3-ol synthesis even further. **g** Introduction of P_*ERG1*_ upstream of *ERG20* in strain MIC3 installs dynamic control of fluxes through Erg20p. In combination with the NPP-specific variants *Cl*LimS(H570Y) and *Sp*SabS(H561F), this leads to further improvement in limonene and sabinene titers, respectively. Strains denoted by GPP express Erg20p(N127W) and produce only GPP, while strains denoted by GPP + NPP express Erg20p(N127W)-*Sl*NPPS1 and produce both GPP and NPP. Samples were prepared independently from three different yeast transformations, processed and analyzed separately (*n* = 3 biologically independent samples). The average yield of each compound between the samples was calculated, and the mentioned ratios and fold improvements in the product profile were deduced. Error bars in panels **a**–**d** correspond to the geometric mean of the original two relative MAD values. Source data of **a**–**d** and **g** are provided as a Source Data file

We explored whether residues analogous to F571 in the other canonical monoterpene synthases play a similarly critical role. Based on amino acid sequence alignment (Supplementary Fig. 13), candidate residues in the other monoterpene synthases were identified and substituted with aromatic or aliphatic side chains of different size using site-directed mutagenesis (Supplementary Table 7). As shown in Fig. 5, in all four enzymes tested, we were able to identify variants at this position that shift substrate selectivity to NPP. Specifically, in *Cl*LimS, all H570 variants showed higher preference for NPP than the wild-type enzyme in the yeast system (Fig. 5a, left panel). Among them, *Cl*LimS(H570Y) and *Cl*LimS(H570F) also achieved improved performance in NPP-based monoterpene production in yeast (Fig. 5a, right panel). In *Cl*LimS(H570Y), this was the result of a twofold improvement on the catalytic efficiency (*k*_cat_^app^/*K*_M_^app^) with NPP, which was mostly due to a lower *K*_M_^app^ with NPP and a decreased *k*_cat_^app^ with GPP (Table 1). In the case of *Cl*LimS(H570F), the higher overall productivity in the yeast system was the result of improved binding of both substrates. *Cl*LimS(H570I), *Cl*LimS(H570L), and *Cl*LimS(H570V) were even more specific for NPP (Fig. 5a, left panel), as manifested by an eightfold higher *k*_cat_^app^/*K*_M_^app^ in *Cl*LimS(H570I) and a threefold higher *k*_cat_^app^/*K*_M_^app^ in *Cl*LimS(H570L) (Table 1). However, the overall efficiency of these variants was low and could not sustain high-product titers (Fig. 5a, right panel; Table 1). We evaluated *Cl*LimS(H570Y) in competition experiments using yeast extracts and ^13^C-NPP. *Cl*LimS(H570Y) was markedly more efficient than wild-type *Cl*LimS in harvesting NPP fluxes, and even when GPP was present at a tenfold excess over ^13^C-NPP, over 75% of the products were derived from ^13^C-NPP (Supplementary Fig. 11b).

In *Sp*SabS, substitution of H561 with F resulted in a highly efficient biocatalyst, with an almost threefold improved apparent specificity for NPP in yeast compared with the wild-type enzyme (Fig. 5b, left panel) and an overall 2.5-fold improvement in performance when the NPP pathway was present (Fig. 5b; right panel). We were not able to obtain active recombinant *Sp*SabS(H561F) to determine the kinetic parameters underlying these improvements. In the case of *Se*CamS, substitution of H583 resulted in drastic changes in specificity and activity. Variants *Se*CamS(H583F) and *Se*CamS(H583V) became limonene-synthesizing enzymes that exhibited three -times higher apparent selectivity for NPP compared to wild-type *Se*CamS (Fig. 5c, left panel). These two variants improved overall NPP-based production in yeast by 11 and 9 times, respectively (Fig. 5c, right panel and Fig. 5e). Finally, in *Pt*PinS, four out of five variants of F607 switched specificity to NPP in the yeast system (Fig. 5d), nevertheless, with lower activity than the parental enzyme. Overall, these results revealed a conserved role for F571 in *Sf*CinS1, and the corresponding residues in the other synthases, in dictating substrate selectivity. The identification of such a key residue in the terpene synthase active site reveals fundamental mechanistic details of these enzymes and will be invaluable for future protein engineering efforts aiming to expand the synthetic pathway developed here with additional synthases.

### Improving titers and enabling downstream oxidation steps

Using the NPP-specific synthases developed here, we were able to harvest the orthogonal pathway established to produce monoterpenes with considerably higher efficiency than only with the GPP-dependent pathway. In shake-flask batch cultivation of strain MIC2 expressing the Erg20p(N127W)-*Sl*NPPS1 fusion, the *Cl*LimS(H570Y)-supported limonene titer reached 130 mg L^−1^, while *Sp*SabS(H561F) enabled the production of 72.7 mg L^−1^ sabinene (Supplementary Table 8). Furthermore, the engineered synthases were quite efficient in harvesting NPP, leading to a significant reduction in nerol production from excess NPP (Supplementary Table 8). Engineering efficient monoterpene scaffold production in yeast is of particular interest because yeast cells offer an advantage over prokaryotic hosts for the functional expression of cytochrome P450 enzymes, which are required for the synthesis of more complex monoterpenoids. So far, low monoterpene titers have hindered efficient P450 catalyzed oxidation of monoterpenes by a yeast cell factory. We evaluated whether the overall improvements obtained here were sufficient to enable downstream oxidation steps by introducing the sabinene hydroxylase CYP750B1^45^ from *T. plicata*, together with a compatible cytochrome P450 oxidoreductase^38,46^, to establish production of *trans*-sabin-3-ol. While production of *trans*-sabin-3-ol was not detectable when only the GPP pathway was used, we were able to synthesize detectable amounts of *trans*-sabin-3-ol in the NPP-producing MIC2 strain using wild-type *Sp*SabS. The levels of *trans*-sabin-3-ol increased three times when the NPP-specific *Sp*SabS(H561F) variant was used instead of *Sp*SabS (Fig. 5f; Supplementary Fig. 14).

### Dynamic regulation of the orthogonal pathway

The metabolic valve created at the point of DMAPP and IPP condensation can be dynamically controlled to divert fluxes towards NPP in response to internal metabolite levels. A key metabolite in our system is ergosterol, the main yeast sterol. To minimize flux to the sterol branch when adequate levels of ergosterol were synthesized, we implemented a dynamic design based on the promoter of the *ERG1* gene. Expression of *ERG1* is negatively regulated by ergosterol via an ergosterol-responsive regulatory element in its promoter (P_*ERG1*_)^47^. A similar design was previously exploited to improve production of the sesquiterpene amorpha-4,11-diene by controlling *ERG9*^48^, and of the diterpene casbene by controlling both the *ERG9* and *ERG20* genes^49^. To dynamically regulate FPP synthesis in response to ergosterol levels in our system, we replaced the promoter of *ERG20* in strain MIC2 with an 807 bp region of P_*ERG1*_ that contains the ergosterol-responsive element to give rise to strain MIC3. Using the engineered NPP-specific synthases and strain MIC3, we were able to produce monoterpenes with considerably higher efficiency than with the native GPP-based pathway. In semi-batch shake-flask cultivation, *Cl*LimS(H570Y)-supported limonene titer in MIC3 strain reached 166 mg L^−1^, which is 6.8-times higher than the titer obtained in GPP-only producing MIC2 cells with wild-type *Cl*LimS (Fig. 5e and Table 2). Similarly, with *Sp*SabS(H561F), we obtained 113 mg L^−1^ sabinene under dynamic regulation (MIC3), which was 7.1 times higher than the highest titer obtained in MIC2 by the native pathway and *Sp*SabS (Fig. 5e and Table 3).

**Table 2:** Production of limonene in semi-batch conditions

Protein expressed	Limonene (mg L^−1^) in MIC2	Nerol (mg L^−1^) in MIC2	Limonene (mg L^−1^) in MIC3	Nerol (mg L^−1^) in MIC3
Erg20p(N127W) + *Cl*LimS wt	23.31 ± 5.97	–	41.49 ± 3.95	–
Erg20p(N127W) + *Cl*LimS(H570Y)	27.51 ± 5.04	–	86.30 ± 10.76	–
Erg20p(N127W)-*Sl*NPPS1 + *Cl*LimS wt	62.47 ± 15.32	0.542	71.84 ± 13.98	0.042
Erg20p(N127W)-*Sl*NPPS1 + *Cl*LimS(H570Y)	126.19 ± 18.97	0.023	167.38 ± 20.88	0.015

Errors correspond to the mean absolute deviation (MAD) around the mean (*n* *=* 3 biologically independent samples). Source data are provided in a Source Data file

**Table 3:** Production of sabinene in semi-batch conditions

Protein expressed	Sabinene (mg L^−1^) in MIC2	Nerol (mg L^−1^) in MIC2	Sabinene (mg L^−1^) in MIC3	Nerol (mg L^−1^) in MIC3
Erg20p(N127W) + *Sp*SabS wt	23.31 ± 5.97	–	41.49 ± 3.95	–
Erg20p(N127W) + *Sp*SabS(H561F)	27.51 ± 5.04	–	86.30 ± 10.76	–
Erg20p(N127W)-*Sl*NPPS1 + *Sp*SabS wt	62.47 ± 15.32	0.542	71.84 ± 13.98	0.042
Erg20p(N127W)-*Sl*NPPS1 + *Sp*SabS(H561F)	126.19 ± 18.97	0.023	167.38 ± 20.88	0.015

Errors correspond to the mean absolute deviation (MAD) around the mean (*n* *=* 3 biologically independent samples). Source data are provided in a Source Data file

## Discussion

We established a synthetic pathway for monoterpenoid synthesis based on an alternative terpene synthase substrate and engineered terpene synthases that preferentially utilize the isomeric substrate. This orthogonal pathway was more efficient than the native GPP-based pathway. Using kinetic studies and competition experiments with ^13^C-labelled substrates, we confirmed that the basis for the improved performance of the NPP-based pathway is that NPP, unlike GPP, is not consumed by Erg20p to produce sterols. Construction of the orthogonal pathway established a metabolic valve at the point of DMAPP and IPP condensation, enabling its dynamic control^4–8,47^. In future efforts, additional titer improvements can be achieved by incorporating more elaborate control mechanisms, such as engineered degradation of Erg20p^23^ or metabolic modeling to identify additional targets for dynamic regulation^9^. This approach has wider implications for the engineering of other high-value compounds where isomers can provide orthogonality, providing a blueprint for applying synthetic pathway design^50^ and orthogonality considerations^3^ in the engineering of biological production systems.

Until now, efforts in monoterpene bioproduction have been more efficient when using bacterial hosts^51–54^. However, engineering the downstream steps of monoterpenoid biosynthesis in a bacterial chassis is challenging because several of these steps require the functional expression of cytochrome P450 enzymes, which is not readily achieved in bacteria^55^. In addition, several monoterpenoids exhibit significant toxicity to bacteria by interfering with the cell membrane, compromising its integrity and impacting the function of membrane-bound enzymes^13,56–58^. Furthermore, accumulation of terpenoid pathway precursors, such as IPP, has been found to have a negative impact on bacterial growth^59^. For these reasons, engineering efficient production of monoterpene scaffolds in yeast can be beneficial. This is highlighted here by the markedly improved production of *trans*-sabin-3-ol using the orthogonal pathway.

These findings also make an important contribution to our understanding of the enzymatic mechanism of monoterpene synthases. The identification of a single residue with a consistent function in dictating isomeric substrate selectivity in the active site of all monoterpene synthases tested points to an evolutionarily conserved role and provide important knowledge for the engineering of NPP-specific monoterpene synthases. This, in turn, will help expand the platform presented here with additional highly efficient synthases for numerous other monoterpene scaffolds. Furthermore, the residue corresponding to *Sf*CinS1(F571) appears to play a multifaceted role in monoterpene synthases, as it has also been found to enable enzymes to differentiate between GPP and 2-methyl-GPP^39^.

The vast majority of known terpenoids are synthesized from substrates with *a trans* configuration. Although the *cis* forms of the universal canonical diterpene precursors Z,Z-FPP and nerylneryl diphosphate (NNPP) have been reported in some organisms, very few *cis*-prenyl diphosphate-derived compounds have been reported so far^28,60–63^. It is unclear what has led to this stereochemical specialization through evolution, but further expansion of the platform developed here to produce *Z,Z*-FPP and NNPP combined with protein engineering of canonical terpene synthases, could provide access to sesquiterpene and diterpenoid compounds that are rare or not found in nature.

## Methods

### Chemicals and enzymes

Standards used include *cis*-3,7-dimethyl-2,6-octadien-1-ol (Aldrich, 268909-5ML), α-pinene (Aldrich, P-7408), β-myrcene (Sigma, M-0382), 1,8-cineole (Aldrich, C8,060-1), γ-terpinene (Aldrich, T2134), limonene (Sigma-Aldrich, 62118), camphene (Sigma-Aldrich, 456055), alpha-phellandrene (Sigma-Aldrich, W285611), 2-carene (Sigma-Aldrich, 232386), beta-phellandrene (Carbosynth, FB158830), linalool (Aldrich, L2602), and a 70% sabinene solution (kindly donated by VIORYL S.A., Athens, Greece). Phusion High-Fidelity DNA Polymerase (New England BioLabs, M0530S) and MyTaq DNA polymerase (BIO-21105, Bioline) were used in PCR amplifications. The QIAquick Gel Extraction Kit (#28704, Qiagen) was used for gel extraction and DNA purification. The NucleoSpin Plasmid Kit (740588.250, Macherey-Nagel) was used for plasmid DNA purification. Restriction enzymes used were from New England BioLabs.

### Yeast media

D-(+)-glucose monohydrate (16301, Sigma); D-(+)-galactose (MG05201, CarboSynth); D(+)-raffinose pentahydrate (OR06197, CarboSynth); Yeast Nitrogen Base w/o AA (Y2025, US Biologicals); Complete Minimal (CM) medium was composed of 0.13% (w/v) dropout powder (all essential amino acids), 0.67% (w/v) yeast nitrogen base w/o AA, 2% glucose; For galactose- based medium, glucose was substituted with 2% galactose and 1% raffinose.

### Construction of MIC3 yeast strain

The promoter region of *ERG1* was amplified from genomic DNA of BY4741 yeast strain using primers SalI-Perg1-FP and Perg1-HindIII-RP to introduce the restriction sites *Sal*I at the 5′-end and *Hind*III at the 3′-end. The PCR product was inserted into a pCR2.1-TOPO vector by TOPO TA Cloning (450641, Thermo Fisher Scientific) and confirmed by sequencing. Subsequently, the pTOPO-P_ERG1_ plasmid was digested with *Sal*I/*Hind*III, and the DNA fragment corresponding to the *ERG1* promoter was gel purified and inserted into pCOD7^24^ linearized with *Sal*I/*Hind*III to generate plasmid COD71 (P_ERG1_-CYC1t, LoxP-HIS5-LoxP). The COD71/P_ERG1_ cassette was PCR amplified using primers COD71-ERG20-FP and COD71-ERG20-RP, adding flanking regions complementary to the 5′ and 3′ end the native promoter of *ERG20* gene. Following transformation into strain MIC2, correct integration was confirmed by PCR using ERG20-gseq-FP and ERG20-gseq-RP.

### Gene cloning and expression in yeast

Supplementary Table 7 contains all primers used in cloning procedures described below.

For cloning of *Sl*NPPS1, the mature form of *Sl*NPPS1 lacking the presumed N-terminal transit sequence (1–44 a.a.) (NM_001247704), was amplified by PCR using primers SlNPPS1-S45BamHI and SlNPPS1-3XhoI. The amplicon was cloned into a bacterial plasmid using the TOPO TA kit (Invitrogen) and validated by Sanger sequencing. *Sl*NPPS1 was subcloned into the yeast vector pHTDH3myc and in the bacterial vector pRSETa using the *Bam*HI and *Xho*I sites.

Fusion of the *Sl*NPPS1 with ERG20(N127W) was initiated by digestion of pYESmyc/ERG20(N127W)-5XGS^14^ with *Eco*RI and *Xho*I to generate a linearized fragment. The *Sl*NPPS1 gene was amplified with SlNPPS1-5EcoRI and SlNPPS1-3XhoI and introduced into pCRII-TOPO vector by TOPO cloning. Subsequently, the *Sl*NPPS1 fragment was released from the above generated construct through restriction digestion with *Eco*RI and *Xho*I. The obtained fragments of pYESmyc/ERG20(N127W)-GS and *Sl*NPPS1 were ligated to generate pYESmyc/ERG20(N127W)-5XGS-*Sl*NPPS1 construct.

Cloning of the TPSs in compatible yeast expression proceeded through the following steps A yeast codon-optimized version of *Citrus limon* (+)-S-limonene synthase (*Cl*LimS) (GenBank AF514287.1) was PCR amplified from pCEV-G2-Ph/*Cl*LimS^64^ using primers LimS-BGL and LimS-XHOstop to introduce *Bgl*II and *Xho*I restriction sites at the 5′ and 3′ ends, respectively. Initially a TOPO cloning reaction was performed to clone the PCR product into vector pCRII-TOPO. Subsequently, the pCRII-TOPO/*Cl*LimS construct was digested using *Bgl*II and *Xho*I restriction enzymes and the *Cl*LimS fragment was excised and subcloned into vector pWTHD3myc^27^ linearized with *Bam*HI and *Xho*I. In this construct, *Cl*LimS was expressed under the control of the P_*TDH3*_ promoter. Yeast codon-optimized versions of *S. lycopersicum* phellandrene synthase (*Sl*PHS1) (FJ797957.1)^28^, *S. habrochaites* limonene synthase (*Sh*LimS) (AFJ67826.1)^30^, *S. habrochaites* pinene synthase (*Sh*PinS) (AFJ67816.1)^30^, and *P. taeda* α-pinene synthase (*Pt*PinS) (GenBank Q84KL3.1)^42^, lacking the transit peptide, were obtained by gene synthesis. In addition, the synthetic fragments bear flanking regions containing specific restriction sites and a generic sequence compatible for USER cloning. The above genes were amplified and cloned by USER cloning into a generic backbone using primers USER-Gen-FP and USER-Gen-RP. *Bam*HI site and *Sal*I sites were introduced at the 5′ and 3′ end, respectively. The constructs were confirmed by sequencing. Subsequently, the genes of interest were excised from the generic USER vector by *Bam*HI and *Sal*I digestion and ligated under the P_GAL1_ promoter into the pESC-TRP vector (Agilent Technologies, Cat. #217453) restricted with *Bam*HI and *Sal*I enzymes. This approach resulted in the construction of the following plasmids: pESC-TRP/*Sl*PHS1, pESC-TRP/*Sh*LimS, pESC-TRP/*Sh*PinS, pESC-TRP/*Pt*PinS. Constructs of *S. fruticosa* 1,8-cineole synthase (*Sf*CinS1) (GenBank ABH07677.1), *S. pomifera* sabinene synthase (*Sp*SabS) (GenBank DQ785794.1) and *Se*CamS in appropriate yeast vectors were available from previous work^14,41,43,65^.

A yeast codon-optimized version of *T. plicata* CYP750B1 (KP004988)^45^ was obtained by gene synthesis, bearing flanking regions containing specific restriction sites and a generic sequence compatible for USER cloning. The corresponding DNA fragment was amplified and cloned into a generic backbone by USER technology using the primers: USER-Gen-FP and USER-Gen-RP (Supplementary Table 7). Subsequently, the gene encoding CYP750B1 was excised from the generic USER vector by *Bam*HI and *Sal*I digestion and ligated under the P_*GAL1*_ promoter into pESC-HIS vector (Agilent Technologies, Cat. #217453) linearized with *Bam*HI and *Sal*I enzymes. The generated plasmid, pESC-HIS/*Tp*CYP750B1, was confirmed by sequencing. A construct (pYX143/ *Pt*CPR2) containing the gene encoding NADPH-cytochrome P450 reductase from *Populus trichocarpa (Pt*CPR2)^46^ was already available^38^.

### Expression in yeast of CYP750B1

Subsequently, MIC2 yeast cells were co-transformed with four expression vectors bearing different auxotrophic markers: pYES2myc for expression of Erg20p(N127W) or Erg20p(N127W)-*Sl*NPPS1 using uracil selection, pJG4-4 for expression of *Sp*SabS wt and mutants (H561F or H561Y) under tryptophan selection, pYX143 for expression of *Pt*CPR2 under leucine selection, and pESC-HIS for expression of *Tp*CYP750B1 using histidine selection. *Pt*CPR2 was expressed from pYX143 (P_TPI1_, *LEU2*, cen) to achieve low level protein expression because this was found to have a positive effect on P450 activity^38^. All other proteins (Erg20p, *Sl*NPPS1, *Sp*SabS, and *Tp*CYP750B1) were expressed under the strong inducible promoter P_*GAL1*_ and from high copy number (2μ) vectors. Sabinene-producing transformants expressing *Tp*CYP750B1/ *Pt*CPR2 were cultured and analyzed by SPME using GC–MS.

### Terpene quantification and extraction from yeast cells

Selected *S. cerevisiae* strains were grown overnight at 30 °C and 150 rpm and subsequently induced in 2-mL liquid media using 20-mL glass vials with magnetic screw cap (Mikrolab Aarhus A/S). Solid phase microextraction (SPME) was applied for measuring the volatile terpenes produced in yeast cells after 48 h of culturing, using a 2 cm-50/30 µm DVB/Carboxen™/PDMS StableFlex™ fiber followed by GC–MS analysis (described below)^65^. For quantification, terpene extraction was performed using 1% (w/v) Diaion HP20 (Supelco, Bellefonte, PA) as adsorbent resin or overlay of the yeast culture with 10% dodecane^14,66,67^ and GC-FID analysis. Prior to addition to the yeast culture, the HP20 resin was activated in 100% methanol. Following culturing, the beads were collected and washed with H_2_O to remove the yeast cells. Subsequently, the beads were eluted three times with ethanol, followed by three pentane elutions including 20–30 min incubation between each step. The pentane phase was initially extracted with equal volume of water and then concentrated to a 100-μL final volume and analyzed. Samples obtained from three separate yeast transformations were processed and analyzed independently.

### Fed batch cultures

Saturated overnight yeast cultures were used to inoculate 10 mL of glucose-based CM selective medium to an OD_600_ = 0.1, which were incubated at 30 °C at 150 rpm shaking until an OD_600_ = 1. The pellets were washed twice with sterile ddH_2_O and resuspended in 10 mL of 2× galactose/raffinose-based CM-selective medium. The cultures were overlaid with 2 mL of dodecane containing 1 ppm isopropyl myristate as internal standard, grown at 30 °C at 150 rpm shaking speed and fed with 1 mL of 20× galactose/raffinose-based CM selective media after 24, 48, 72, and 96 h. Cultures were stopped after 6 days of cultivation and the dodecane phase was collected and diluted 1:100 in hexane containing 1 ppm squalene internal standard. Following GC-FID analysis, samples were quantified by comparison of retention time and peak area to that of standard curves of limonene and sabinene authentic standard.

### Enzyme expression and purification

Enzymatic activity of terpene synthases, *Sl*NPPS1 and Erg20p with GPP and NPP was evaluated with *E. coli*-produced and purified recombinant enzyme and with yeast cell extracts. Wild-type and mutant 6×His-tagged TPSs^39,41^, *Sl*NPPS1^28^, and Erg20p^14,37^ were purified by Ni^2+^-NTA affinity chromatography from 200 mL cultures of *E. coli* BL21 DE3 growing in LB media at 19 °C^14,37,39^. Protein expression was induced by addition of 0.1 mM IPTG the cells were grown overnight at 19 °C. Subsequently, protein purification was performed by affinity chromatography using Ni-NTA agarose resin (Qiagen). Briefly, cells were disrupted by sonication in lysis buffer (60 mM Tris, pH 8, 20 mM *β*-mercaptoethanol, 0.35 M NaCl, 10 mM imidazole, 10% (w/v) glycerol, 1 mM PMSF, 1 mg/mL lysozyme)^41^. Following removal of the cell debris via centrifugation 2400 × *g* and 4 °C, the protein lysate was incubated with Ni-NTA agarose resin for 1 h at 4 °C with mild shaking. The protein was washed twice and eluted from the resin using 350 mM imidazole.

### Enzymatic assays

Terpene synthase activity was assayed in a 0.5-mL reaction containing 20 mM MOPS (pH 7.0), 20 mM MgCl_2_, 0.02 mM MnCl_2_, 1 mM DTT, 0.1 mg mL^−1^ BSA, 50 ng enzyme and varying concentrations (0–150 µM) of substrate (GPP or NPP). The reaction was overlaid with an equal volume of hexane containing 1 ppm nonane and 1 ppm dodecane as internal standards and incubated for 1 h at 30 °C. The hexane phase was recovered and 2 μL were analyzed by GC–MS^9,41^. Prenyl diphosphate synthase activity was assayed in a 0.2-mL reaction containing 10 mM MOPS (pH 7.0), 5 mM MgCl_2_, 1 mM DTT, 0.1 mg mL^−1^ BSA, 0.02 mM IPP, and 50 ng of recombinant Erg20p. GPP or NPP substrates were added at 0.02 mM. Reactions were terminated by addition of an equal volume (0.2 mL) of 2 N HCl in 83% ethanol and 0.2 mL of hexane. Following a 20 min incubation at 37 °C to allow diphosphate hydrolysis, reactions were neutralized with 0.2 mL of 10% NaOH. A 2-μL aliquot of the hexane phase of an in vitro enzymatic assay was analyzed by GC–MS. *Sl*NPPS1 enzymatic activity was assayed in the same conditions as for Erg20p^11^, using 50 ng of purified enzyme, 0.05 mM of IPP or DMAPP, and varying concentrations (0–0.2 mM) of DMAPP or IPP, respectively. Reactions were incubated for 1 h at 30 °C and stopped by acid treatment. Kinetic analysis was performed using WinCurveFit 1.1.8 (Kevin Raner software). Yeast cells obtained from a 10-mL culture were resuspended in 10 mM MOPS (pH 7.0), 5 mM MgCl_2_, and 1 mM DTT and disrupted by glass beads. The resulted yeast extracts were used in enzymatic assays as described above for bacterial purified terpene synthases and prenyl diphosphate synthases.

### Competition assays

Yeast extracts obtained as described above from cells expressing *Sl*NPPS1, Erg20p, *Cl*LimS wild-type or *Cl*LimS(H570Y) were used in 0.5-mL reaction containing 20 mM MOPS, 20 mM MgCl_2_, 0.02 mM MnCl_2_, 1 mM DTT, 0.01% (v/v) BSA, and varying concentrations of IPP, DMAPP, GPP, NPP, ^13^C-GPP, or ^13^C-NPP. The reactions were overlaid with hexane containing 1 ppm nonane and 1 ppm dodecane as internal standards and incubated for 1 h at 30 °C. Parallel reactions for hexane extraction and acid treatment following hexane extraction were performed^14,37^. The hexane phase (2 μL) was analyzed by GC-FID for product quantification and by GC–qTOF for detection of the ^13^C isotope incorporated in reaction products.

### Yeast growth assay

AM94 cells harboring the pTDH3myc/*Sl*NPPS1 construct or the empty pTDH3myc vector were grown in glucose-based media at 30 °C and 150 rpm for 20 h. Yeast growth was monitored by measuring the optical density at 600 nm at regular intervals. No difference in the growth curve was observed in yeast cells expressing *Sl*NPPS1 under the P_*TDH3*_ constitutive promoter in comparison to cells carrying the empty vector.

### Toxicity assays

Two different assays were considered to evaluate the limitation imposed on monoterpene production in yeast cells due to possible toxicity exhibited by these molecules to *S. cerevisiae*. The selected monoterpenes were limonene and sabinene. To assess the effect of monoterpene on yeast growth, AM94 yeast cells were used to inoculate 20 mL media to OD_600_ ~0.05 (considered time 0). The cultures were overlaid with dodecane treated with varying concentrations of limonene or sabinene, respectively, to a final concentration of 0, 30, 100, and 500 mg L^−1^ and incubated at 30 °C with 150 rpm shaking. The yeast cellular growth was monitored by OD_600_ measurements taken every 2 h over a period of time of 34 h. For OD_600_ values higher than 1, samples were diluted accordingly.

To evaluate the effect of limonene accumulation, yeast cells expressing *Cl*LimS (100 µL) were used to inoculate 10 mL of galactose/raffinose-based CM-selective media overlaid with 10% dodecane containing varying concentration of limonene (0 mg L^−1^, 300 mg L^−1^, and 1 g L^−1^) and incubated for 48 h at 30 °C with 150 rpm shaking. Subsequently, the dodecane phase was recovered as described above and analyzed by GC-FID. The limonene exceeding the starting concentration was considered to be synthesized by yeast cells.

### GC–MS analysis

GC–MS analysis was carried out using a ZB-5ms column and helium as a carrier gas with a constant velocity of 37 cm/s. Samples resulting from incubation of the SPME fiber for 30 min over the head space of the yeast cultures were analyzed using the following temperature program: initial temperature 60 °C, ramp to 135 °C with a rate of 3 °C min^−1^, ramp to 240 °C with a rate of 20 °C min^−1^, and hold for 5 min. To detect terpenols derived by the acid treatment of prenyl diphosphates, the temperature program of GC–MS analysis was modified as follows: initial temperature 60 °C, hold for 3 min, ramp to 80 °C with a rate of 3 °C min^−1^, ramp to 110 °C with a rate of 30 °C min^−1^, ramp to 130 °C with a rate of 3 °C min^−1^, ramp to 280 °C with a rate of 30 °C min^−1^ and hold for 3 min.

### GC-FID analysis

GC-FID was carried on a HP-5MS UI capillary column (Agilent Technologies) with a (5%-Phenyl)-methylpolysiloxane (Ultra Inert) stationary phase. Column dimensions were 30 m length × 0.25 mm internal diameter × 0.25 film thickness. Helium was used as a carrier gas with a constant velocity of 50 cm/s. The instrument was equipped with a PTV injector. Hexane samples were analyzed using the following temperature program: hold initial temperature at 40 °C for 3 min, ramp to 80 °C with a rate of 3 °C min^−1^, followed by a ramp to 300 °C with a rate of 30 °C min^−1^, and a hold for 10 min.

### GC–APCI–QqToF analysis

GC–APCI–QqToF analyses were performed using a Scion 456 GC system (Bruker Daltonics) coupled to a micrOTOF-Q II (QqToF) mass spectrometer (Bruker Daltonics) via an atmospheric pressure chemical ionization (APCI) source. Samples were analyzed by injecting 1 µL aliquots in splitless injection mode (injector temperature 250 °C) onto a BR-5ms capillary column (30 m × 0.25 mm ID × 0.25 µm film thickness; Bruker Daltonics). The samples were eluted using helium as the carrier gas with a constant flow of 1.0 mL min^−1^. The temperature gradient of the GC oven was programmed as follows: initial temperature 50 °C was held for 1 min, before a ramp to 280 °C at a rate of 15 °C min^−1^, and subsequent hold for 3.67 min, giving a total method duration of 20 min. The transfer line between the GC and QqToF was maintained at 280 °C throughout the analysis. The head temperature of the APCI source was set to 280 °C, and the APCI heater to 200 °C, with a charging voltage of 2000 V. The QqToF mass spectrometer was operated in full scan positive ion mode with the following instrument settings: *m/z* 80–310: nebulizer gas (nitrogen), 3.5 bar; drying gas (nitrogen), 2.5 L min^−1^; drying gas temperature, 240 °C; capillary voltage, 3000 V; spectra acquisition rate, 5 Hz. Every chromatogram was calibrated for accurate mass using automated infusion of perfluorotributylamine vapor (PFTBA; Sigma-Aldrich) for a short period at the beginning of each chromatographic run. All data acquisition was automated using a combination of Compass CDS (Version 3.0.1; Bruker Daltonics), Compass oTOF Control (Version 3.4, Bruker Daltonics) and Hystar (Version 3.2 SR4, Bruker Daltonics) software. For data analysis, Compass DataAnalysis software (Version 4.3, Bruker Daltonics) was used.

### Calculation of labeled:unlabeled limonene ratio

The relative contribution of GPP and NPP to limonene synthesis was estimated in competition assays where either GPP or NPP was labeled with two ^13^C, causing a +2 mass shift in the proportion of the limonene mass spectra stemming from the labeled substrate. The limonene mass spectra contained a large amount of −2H ions for each major ion, causing the ^13^C-labeled limonene to have significant overlap with the major ions of unlabeled limonene. The ratio between unlabeled and labeled limonene could, therefore, not be accurately calculated directly from the + 2 shift in major mass ions. It was instead estimated by comparing four ion pairs of unlabeled −2H and labeled +2^13^C of *m/z* 135/139, 166/170, 191/195 and 205/209. The ratios for each pair were calculated based on the known relative ratio between signal intensities of each ion pair in the mass spectra between pure unlabeled and labeled limonene at equal concentrations. The weighted average and standard errors were calculated using the signal to noise for each pair as weighting. Details with exact numbers and calculations are provided in the Data Source file (sheets named Supplementary Fig. 12a and Supplementary Fig. 12b).

### Molecular graphics

Molecular graphics were produced using the UCSF Chimera package. Chimera is developed by the Resource for Biocomputing, Visualization, and Informatics at the University of California, San Francisco (supported by NIGMS P41-GM103311)^68^.

### Compound identification

Identification of monoterpene compounds produced by yeast cells was based on GC–MS analysis and comparison of retention times and mass spectra with authentic standards (listed earlier in this section under Chemicals and enzymes).

### Mutagenesis

Site-directed mutagenesis using the Quickchange method (Agilent) was performed for *Sf*CinS1, *Sp*SabS, and *Sel*CamS, while USER mutagenesis^69^ was performed for *Pt*PinS and *Cl*LimS. The primers used for mutagenesis purposes are listed in Supplementary Table 7.

### Construction of the *Sf*CinS model

A structural model of *Sf*CinS1 was constructed using the SWISS-MODEL server^70^ using mint limonene synthase (pdb id: 2ong) as template. The 2-fluorolinalyl diphosphate and 2-fluorogeranyl diphosphate ligands were then superimposed on the model, based on their position in the mint limonene synthase structure.

### General chemical synthesis procedures

All reactions were monitored by TLC on aluminum sheets coated with silica gel 60F254 (0.2-mm thickness, Merck) and the components present were detected by charring with 10% H_2_SO_4_ in MeOH. Column chromatography was carried out using silica gel 60 (particle size 0.040–0.063 mm, 230–400 mesh ASTM, Merck). Solvent extracts were dried with anhydrous MgSO_4_ unless otherwise specified. The ^1^H and ^13^C NMR spectra were recorded on a Bruker Avance 400 spectrometer at 400 and 101 MHz, respectively. CDCl_3_ was used as solvent (unless otherwise indicated), δH values are relative to internal TMS and δC values are referenced to the solvent [δC (CDCl_3_) = 77.0].

### Synthesis of ^13^C-labeled neryl- and GPP

Chemical synthesis of (Z)-[1,2-^13^C_2_]-neryl pyrophosphate **5a** and its isomer (E)-[1,2- ^13^C_2_]-geranyl pyrophosphate **5b** was performed according to the following procedure:

To synthesize ethyl (*Z*)-[1,2-^13^C_2_]-3,7-dimethylocta-2,6-dienoate (**3a**) and ethyl (*E*)-[1,2-^13^C_2_]-3,7-dimethylocta-2,6-dienoate (**3b**), a neat mixture of LiCl (0.25 g, 5.9 mmol), triethyl phosphono-^13^C_2-_acetate (**1**) (1.0 g, 4.4 mmol) and DBU (1,8-diazabicyclo[5,4,0]undec-7-ene) (0.72 mL, 4.9 mmol) was stirred at rt for 1.0 h under Ar followed by the addition of 6-methyl-5-hepten-2-one (**2**) (2.3 mL, 15.6 mmol). Stirring was continued at rt for 24 h. The reaction was quenched with water (50 mL) and the reaction mixture was extracted with Et_2_O (200 mL). The extract was washed with brine and dried (MgSO_4_). The solvent was evaporated and chromatographed on silica (100 g) with 0–10% Et_2_O in n-pentane to afford the separation of the pure (*Z*)-isomer **3a** as colorless oil (87.0 mg, 10%), *E*/*Z*-mixture (560.0 mg, 68%) and pure (*E*)-isomer **3b** (175.0 mg, 20%) (829.0 mg in total, 95%).

To synthesize (*Z*)-[1,2-^13^C_2_]-3,7-dimethylocta-2,6-diene-1-ol (**4a**), a stirred solution of ethyl (*Z*)-[1,2- ^13^C_2_]-3,7-dimethylocta-2,6-dienoate (**3a**) (60.0 mg, 0.30 mmol) in diethyl ether (5.0 mL) and cooled to −78 °C. DIBAL-H (1.0 M in toluene) (0.73 mL, 0.73 mmol) was added dropwise and the reaction mixture was stirred at −78 ***°***C for 1 h and then quenched by addition of MeOH (0.5 mL). The reaction mixture was warmed gradually to 0 °C and diluted with Et_2_O (10 mL) followed by the addition of saturated aqueous solution of Rochelle salt (10 mL), water (10 mL) and vigorously stirred. Once the Et_2_O layer became clear, it was separated, dried over MgSO_4_, filtered, and concentrated on a rotary evaporator under reduced pressure. Chromatographic purification of the resulting oil on silica (14 g) with 20% Et_2_O in n-pentane afforded the separation of the pure **4a** colorless oil (45.0 mg, colorless oil, 96%).

(*E*)-[1,2-^13^C_2_]-3,7-dimethylocta-2,6-diene-1-ol (**4b**) was synthesized exactly as described for the alcohol **4a**.

The structure of (*Z*)- [1,2-^13^C_2_]-3,7-Dimethylocta-2,6-diene-1-ol (**4a**) was confirmed by NMR and HRMS analysis. ^1^H NMR (600 MHz, CDCl_3_): δ1.61 (s, 3H), 1.69 (d, ^4^*J*_H,H_ *=* 0.9 Hz,3H), 1.75 (dd, ^3^*J*_CH3,C-1_ *=* 6.0 Hz, ^4^*J*_H,H_ *=* 0.9 Hz, 3H), 2.10 (m, 4H,–C*H*_*2*_C*H*_*2*_–), 3.42 (d, ^2^*J*_OH,C-1_ = 6.5 Hz,1H, OH), 4.09 (dddd, ^1^*J*_H-1,C-1_ * =* 141.9 Hz, ^2^*J*_H-1,H-1_ *=* 12.3 Hz, ^3^*J*_H-1,H-2_ *=* 7.2 Hz, ^2^*J*_H-1,C-2_ *=* 4.0 Hz, ^4^*J*_H-1,H-4_ *=* 0.8 Hz, 2H, –^13^C*H*_*2*_–OH), 5.10 (m, 1H, H-6), 5.45 (dtm, ^1^*J*_H-2,C-2_ *=* 152.4 Hz, ^3^*J* *=* 7.2 Hz, 1H); ^13^C NMR (151 MHz, CDCl_3_): δ 59.2 (d, ^1^*J*_C–C_ = 47.5 Hz, ^13^C-1), 124.4 (d, ^1^*J*_C–C_ = 47.5 Hz, ^13^C-−2), (only peaks for ^13^C-labeled carbons reported). HRMS (m/z): [M + H–H_2_0] + calcd. For ^13^C_2_C_8_H_18_O, 139.1397; found, 139.1392.

The structure of (*E*)-[1,2-^13^C_2_]-3,7-Dimethylocta-2,6-diene-1-ol (**4b**) was confirmed by NMR and HRMS analysis. H NMR (600 MHz, CDCl_3_): δ1.61 (s, 3H), 1.68 (s, 3H), 1.75 (s, 3H), 2.03 and 2.11 (2m, 4H,–C*H*_*2*_C*H*_*2*_–), 3.41 (d, ^2^*J*_OH,C−1_ = 6.5 Hz,1H, OH), 4.16 (dddd, ^1^*J*_H-1,C-1_ *=* 141.8 Hz, ^2^*J*_H-1,H-1_ *=* 11.5 Hz, ^3^*J*_H-1,H-2_ *=* 7.0 Hz, ^2^*J*_H-1,C-2_ *=* 4.0 Hz, ^4^*J*_H-1,H-4_ *=* 0.6 Hz, 2 H, –^13^C*H*_*2*_–OH), 5.10 (tm, *J* *=* 7.0 Hz,1H, H−6), 5.45 (dtm, ^1^*J*_H−2,C−2_ *=* 152.4 Hz, ^3^*J* *=* 7.0 Hz, 1H). ^13^C NMR (151 MHz, CDCl_3_): δ59.6 (d, ^1^*J*_C–C_ = 47.5 Hz, ^13^C-1), 124.4 (d, ^1^*J*_C–C_ = 47.5 Hz, ^13^C-2), (only peaks for ^13^C-labeled carbons reported). HRMS (*m*/*z*): [M + H–H_2_0] + calcd. For ^13^C_2_C_8_H_18_O, 139.1397; found, 139.1387.

To synthesize (*Z*)-[1,2-^13^C_2_]-neryl pyrophosphate (**5a)**, a stirred solution of (Z)-[1,2-^13^C_2_]-3,7-dimethylocta-2,6-diene-1-ol (**4a**) (15.6 mg, 0.10 mmol) in dry benzene (2.0 mL) and dry CH_2_Cl_2_ (0.2 mL) was added CBr_4_ (68.0 mg, 0.21 mmol) and Ph_3_P (54.0 mg, 0.21 mmol) at 0 °C under a nitrogen atmosphere. After the solution was stirred at the same temperature for 2 h, n-pentane (2.0 mL) was added to the reaction mixture. The reaction mixture was filtered to remove triphenylphosphine oxide. The filtrate was concentrated under reduced pressure to afford the crude (*Z*)-neranyl bromide (22.0 mg, quant.) as yellow oil which was immediately subjected to the following pyrophosphorylation reaction without further purification. A solution of the crude bromide in dry CH_3_CN (2 mL) was added dropwise to a stirred solution of [(n-Bu_4_)N]_3_HP_2_O_7_ (135.0 mg, 0.15 mmol, 1.5 eq) in dry CH_3_CN (2.0 mL) at 0 °C under Ar. The mixture was stirred at rt for 24 h and concentrated on a rotary evaporator below 35 °C. The obtained residue was transferred to a centrifuge tube with 5.0 mL of acetone and concentrated NH_4_OH (0.5–1 mL) was added. The precipitated ammonium salts, isolated by centrifugation (1500 × *g* for 10 min), washed twice by resuspension in 5.0 of acetone containing 0.01 N NH_4_OH. The combined supernatants of the acetone containing NH_4_OH were roto-evaporate at below 35 °C. The resulting crude (*Z*)-[1,2-^13^C_2_]-neryl pyrophosphate **5a** as its ammonium salt was pure enough to be used for the enzymatic studies. However, a pure sample of **5a** was accomplished by preparative TLC-plates using the eluent system MeOH/CH_2_Cl_2_/0.01 N NH_4_OH (1: 1.2: 0.3 v/v).

(*E*)-[1,2-^13^C_2_]-geranyl pyrophosphate **5b** was synthesized exactly as described for **5a**. When synthesized organic pyrophosphates **5a** and **5b** were subjected to acid hydrolysis, the recovered alcohol was compared with authentic samples by TLC and GC–HRMS. In each case, the recovered alcohol was essentially identical with the corresponding alcohols **4a** and **4b**, respectively.

### Reporting summary

Further information on research design is available in the Nature Research Reporting Summary linked to this article.

## Supplementary information

Supplementary Info

Peer Review

Reporting Summary

Source Data

## Data Availability

Data supporting the findings of this work are available within the paper and its Supplementary Information files. A reporting summary for this article is available as a Supplementary Information file. The data sets generated and analyzed during this study are available from the corresponding author upon request. The source data underlying Tables 1–3; Figs. 2c, d, 3a–e, 4a, 5a–d, g; Supplementary Tables 2–6, and 8; Supplementary Figs. 1, 2, 3a, b, 4a, b, 6, 7, 8a, b, 10a, b, 12a, b are provided as a Source Data file.
