# Supplementary material for: Orthogonal monoterpenoid biosynthesis in yeast constructed on an isomeric substrate
Source: Nat Commun. 2019 Aug 23;10:3799. doi: 10.1038/s41467-019-11290-x (PMC6707142; doi:10.1038/s41467-019-11290-x)
Supplement: Supplementary file 1 — Supplementary Info [file 41467_2019_11290_MOESM1_ESM.pdf]

SUPPLEMENTARY INFORMATION

Orthogonal monoterpenoid biosynthesis in yeast constructed on an isomeric substrate

Ignea *et al.*

Table of contents

SUPPLEMENTARY TABLES

Supplementary Table 1. List of the yeast strains used in this study.
Supplementary Table 2. Overview of monoterpene yield improvement during optimization of the NPP-producing platform.
Supplementary Table 3. Overview of nerol production by yeast cells expressing the NPP-specific monoterpene synthases *Sl*PHS1, *Sh*LimS, and *Sh*PinS.
Supplementary Table 4. Kinetic parameters of *Sl*NPPS1 and Erg20p.
Supplementary Table 5. Monoterpene production by NPP-specific versus canonical enzymes.
Supplementary Table 6. Monoterpene compound titer obtained with the different *Sf*CinS1 mutants expressed as percentage of the titer obtained with wild-type *Sf*CinS1.
Supplementary Table 7. List of primers and mutants
Supplementary Table 8. Overview of limonene, sabinene and nerol production in engineered yeast strains under shake-flask batch cultivation conditions.

SUPPLEMENTARY FIGURES

Supplementary Figure 1. Evaluation of the effect of monoterpenes in the growth of AM94 yeast cells.
Supplementary Figure 2. The presence of the monoterpene product does not inhibit further production of limonene by the yeast cells.
Supplementary Figure 3. Evaluation of substrate flux.
Supplementary Figure 4. SDS-PAGE of the recombinant proteins purified from *E. coli* and used in the *in vitro* assays.
Supplementary Figure 5. Evaluation of the ability of yeast cells to use NPP as a substrate for the production of larger diphosphates.
Supplementary Figure 6. Growth curves of yeast cells overexpressing *Sl*NPPS1 or containing the empty vector.
Supplementary Figure 7. Western blot analysis of myc-tagged *Sl*NPPS1, Erg20p(N127W) and two fusion proteins in different orientation, Erg20p(N127W)-*Sl*NPPS1 and *Sl*NPPS1-Erg20p(N127W) fusion.
Supplementary Figure 8. In vitro and in vivo product profiles of the canonical terpene synthases.

Supplementary Figure 9. Chemical synthesis of (Z)- [1,2- $^{13}C_2$]-neryl pyrophosphate **5a** and (E)- [1,2-$^{13}C_2$]-geranyl pyrophosphate **5b**.
Supplementary Figure 10. NPP is used exclusively for the production of monoterpenes and not consumed for the synthesis of larger prenyl diphosphates.
Supplementary Figure 11. Mass spectra of the products synthesized in enzymatic reactions of yeast extracts containing *Cl*LimS with labeled and unlabeled substrates.
Supplementary Figure 12. Substrate competition experiment.
Supplementary Figure 13. Alignment of the plant terpene synthases used in this study showing the residues corresponding to F571 in *Sf*CinS1.
Supplementary Figure 14. Mass spectrum of the *trans*-sabin-3-ol product obtained in yeast cells expressing *Sl*NPPS1, *Sp*SabS(H561F) and CYP750B1.

REFERENCES

SUPPLEMENTARY TABLES

Supplementary Table 1. List of the yeast strains used in this study.

**Strain**	**Genotype**	**Source**
AM94	Mat a/α, P_{GAL1}-*HMG2*(K6R):: HOX2, *ura3*, *trp1*, *his3*, P_{TDH3}-*HMG2*(K6R)::*leu2X*2, *ERG9/erg9*	Ref.[1]
MIC2	Mat a/α, P_{GAL1}-*HMG2*(K6R)::HOX2, *ura3*, *trp1*, *his3*, P_{TDH3}-*HMG2*(K6R)-::*leu2X2*, *ERG9/erg9*, *ERG20/erg20*	Ref.[2]
MIC3	Mat a/α, P_{GAL1}-*HMG2*(K6R)::HOX2, *ura3*, *trp1*, *his3*, P_{TDH3}-*HMG2*(K6R)-::*leu2X2*, *ERG9/erg9*, P_{ERG1}-*ERG20/erg20*	This study

Supplementary Table 2. Overview of monoterpene yield improvement during optimization of the NPP-producing platform.

	**Strain**	**Protein expressed**	**Titer (mg/L)**	**Fold improvement (from previous step)**	**Fold improvement (total)**
**Production of β-phellandrene by *Sl*PHS1**	AM94	*Sl*NPPS1	0.159 ± 0.015	-	-
	AM94	*Sl*NPPS1 + Erg20p(N127W)	0.673 ± 0.042	4.226	4.226
	MIC2	*Sl*NPPS1 + Erg20p(N127W)	1.897 ± 0.318	2.817	11.904
	MIC2	Erg20p(N127W)-*Sl*NPPS1	2.700 ± 0.313	1.424	16.946
**Production of limonene by *Sh*LimS**	AM94	*Sl*NPPS1	0.135 ± 0.045	-	-
	AM94	*Sl*NPPS1 + Erg20p(N127W)	0.447 ± 0.154	3.307	3.307
	MIC2	*Sl*NPPS1 + Erg20p(N127W)	1.201 ± 0.400	2.685	8.879
	MIC2	Erg20p(N127W)-*Sl*NPPS1	2.480 ± 0.827	2.065	18.334
**Production of α-pinene by *Sh*PinS**	AM94	*Sl*NPPS1	0.009 ± 0.001	-	-
	AM94	*Sl*NPPS1 + Erg20p(N127W)	0.028 ± 0.003	3.282	3.282
	MIC2	*Sl*NPPS1 + Erg20p(N127W)	0.055 ± 0.008	1.953	6.409
	MIC2	Erg20p(N127W)-*Sl*NPPS1	0.110 ± 0.015	1.984	12.719

Three NPP-specific terpene synthases were evaluated throughout the different interventions: *Sl*PHS1, *Sh*LimS and *Sh*PinS. Errors correspond to the mean absolute deviation (MAD) around the mean (n = 3 biologically independent samples resulting from different yeast transformations). Source data are provided as a Source Data file.

Supplementary Table 3. Overview of nerol production by yeast cells expressing the NPP-specific monoterpene synthases *Sl*PHS1, *Sh*LimS, and *Sh*PinS.

**Strain**	**Protein expressed**	**Nerol titer (mg/L)**		
		**Cells expressing *Sl*PHS1**	**Cells expressing *Sh*LimS**	**Cells expressing *Sh*PinS**
AM94	*Sl*NPPS1	0.119 ± 0.014	0.136 ± 0.045	0.263 ± 0.078
AM94	*Sl*NPPS1 + Erg20p(N127W)	0.773 ± 0.031	0.822 ± 0.274	1.410 ± 0.093
MIC2	*Sl*NPPS1 + Erg20p(N127W)	1.497 ± 0.131	1.124 ± 0.376	3.720 ± 0.327
MIC2	Erg20p(N127W)-*Sl*NPPS1	2.857 ± 0.222	2.407 ± 0.813	4.460 ± 0.567

Errors correspond to the mean absolute deviation (MAD) around the mean. *n* = 3 biologically independent samples (separate yeast transformations). Source data are provided as a Source Data file.

Supplementary Table 4. Kinetic parameters of *Sl*NPPS1 and Erg20p.

Enzyme variant	Substrate	k_{cat}^{app} (min^{-1})	K_M^{app} (μM)	k_{cat}^{app}/K_M^{app} (min^{-1} μM^{-1})	Source
*Sl*NPPS1	DMAPP	0.037 ± 0.001	66.72 ± 6.32	0.499 x10^{-3}	This study
Erg20p wild-type	DMAPP	0.025 ± 0.014	0.17 ± 0.08	147 x10^{-3}	Ref.[3]
Erg20p (N127W)	DMAPP	0.010 ± 0.004	0.30 ± 0.08	33.33 x10^{-3}	Ref.[3]
*Sl*NPPS1	IPP	0.041 ± 0.003	83.69 ± 17.41	0.564 x10^{-3}	This study
Erg20p wild-type	GPP	0.061 ± 0.024	0.43 ± 0.07	0.141	Ref.[3]
Erg20p (N127W)	GPP	0.011 ± 0.005	22.61 ± 3.51	0.486	Ref.[3]

Kinetic parameters of *Sl*NPPS1 determined as described in the Methods section. Kinetic parameters of Erg20p and Erg20p(N127W) were determined as described in Ignea *et al*., (2014). Errors represent standard error of the fitted parameters (*n* = 3 technical replicates). Source data are provided as a Source Data file.

Supplementary Table 5. Monoterpene production by NPP-specific versus canonical enzymes.

Enzyme	GPP	GPP+NPP
***Sh*PinS**	0.00±0.00	0.11±0.01
***Pt*PinS**	9.15±1.30	24.59±3.52
***Sh*LimS**	0.00±0.00	2.48±0.05
***Cl*LimS**	27.97±2.41	64.64±7.55

Errors for each sample, corresponding to the mean absolute deviation (MAD) around the mean are listed below for clarity. $n = 3$ biologically independent samples. Source data are provided as a Source Data file.

Supplementary Table 6. Monoterpene compound titer obtained with the different *Sf*CinS1 mutants expressed as percentage of the titer obtained with wild-type *Sf*CinS1.

	***Sf*CinS1 (F571H)**	***Sf*CinS1 (F571Y)**	***Sf*CinS1 (F571V)**	***Sf*CinS1 (F571L)**	***Sf*CinS1 (F571I)**
**% of wild-type yield** with GPP only	0.4±0.11	0.21±0.1	0.2±0.12	0.23±0.11	0.2±0.10
**% of wild-type yield** with GPP and NPP	1.42±0.2	1.86±0.3	2.59±0.5	0.14±0.09	2.40±0.8

Errors correspond to the mean absolute deviation (MAD) around the mean (*n* = 3 biologically independent samples - different yeast transformation processed and analyzed separately). Source data are provided as a Source Data file.

Supplementary Table 7. List of primers and mutants.

Gene Name	Primer	Sequence	Mutation
*Sl*NPPS1	SlNPPS1-S45BamHI	5'- ggatccatgtctgctcgtggactcaacaagatttcatgct -3'	
	SlNPPS1-3XhoI	5'- ctcgagtcaatatgtgtgtccaccaaaacgtctatgcctt -3'	
	SlNPPS1-5EcoRI	5'- gaattcatgtctgctcgtggactcaacaag -3'	
*Cl*LimS	LimS-BGL	5'- agatct-atgagaagatcagctaactatcaacc -3'	
	LimS-XHOstop	5'- ctcgag-tcaaccctttgtacctggtgatg -3'	
USER generic	USER-Gen-FP	5'- cacgcgauaagtccggtacgaattcggatcc -3'	
	USER-Gen-RP	5'-cgtgcgaugcggccgcagatctgtcgac-3'	
*Sf*CinS1	CinS-F571VLI	5'- tgaatgttgcatgccaavtccatcagattcatgc -3'	F571V; F571L; F571I
	CinS-L485AG	5'- gggcgtcgtgtactattctagstaggcttgctgatgatatggg	L485A; L485G
	CinS-L556AG	5'- attgcgccattctggcascattagcagaaacttctacaaaata -3'	L556A; L556G
	CinS-Y-U-FP	5'- atatggcaugcaacattcattggtc -3'	N338S-F571Y
	CinS-Y-U-RP	5'- atgccatauccatcagattcatgctggtatatc -3'	
	CinS-H-U-FP	5'- acatggcaugcaacattcattggtc - 3'	N338I-F571H; N338A-F571H; N338C-F571H; I451V-F571H; N338S-F571H
	CinS-H-U-RP	5'- atgccatguccatcagattcatgctggtatatc - 3'	
	CinS-V-U-FP	5'- agttggcaugcaacattcattggtc - 3'	N338S-F571V
	CinS-V-U-RP	5'- atgccaacuccatcagattcatgctggtatatc - 3'	
	CinS-L-U-FP	5'- acttggcaugcaacattcattggtc - 3'	N338S-F571L
	CinS-L-U-RP	5'- atgccaaguccatcagattcatgctggtatatc - 3'	
	CinS-I-U-FP	5'- aattggcaugcaacattcattggtc - 3'	N338S-F571I
	CinS-I-U-RP	5'- atgccaatuccatcagattcatgctggtatatc - 3'	
*Sel*CamS	CamS-H583FY	5'- ctcataattttgaattccgwatccatctccatgctg -3'	H583F; H583Y
	CamS-H583VLI	5'- cataattttgaattccgavtccatctccatgctg -3'	H583V; H583L; H583I
*Sp*SabS	SabS-H561FY	5'- cgagtgttgcacgccawacccatccccgtcgag -3'	H561F; H561Y
	SabS-H561VLI	5'- cgagtgttgcacgccaavcccatccccgtcgag -3'	H561V; H561L; H561I
*Cl*LimS	LimS H570F U FP	5'- acggtttcggugtacaaaatcaagaaacca -3'	H570F
	LimS H570F U RP	5'- accgaaaccgucaccatgcaaatacatga -3'	
	LimS H570Y U FP	5'- acggttacggugtacaaaatcaagaaacca -3'	H570Y
	LimS H570Y U RP	5'- accgtaaccgucaccatgcaaatacatga -3'	
	LimS H570V U FP	5'- acggtgtcggugtacaaaatcaagaaacca -3'	H570V
	LimS H570V U RP	5'- accgacaccgucaccatgcaaatacatga -3'	
	LimS H570L U FP	5'- acggtcucggtgtacaaaatcaagaaacca -3'	H570L
	LimS H570L U RP	5'- agaccgucaccatgcaaatacat -3'	
	LimS H570I U FP	5'- acggtatcggugtacaaaatcaagaaacca -3'	H570I
	LimS H570I U RP	5'- accgataccgucaccatgcaaatacatga -3'	
*Pt*PinS	PtPinS-F607H-F	5'- atggtcacuctgttgcttccatcgaaatc -3'	F607H
	PtPinS-F607H-R	5'- agtgaccauctctgtacttgtaaccgtagt -3'	
	PtPinS-F607Y-F	5'- atggttacuctgttgcttccatcgaaatc -3'	F607Y
	PtPinS-F607Y-R	5'- agtaaccauctctgtacttgtaaccgtagt -3'	
	PtPinS-F607V-F	5'- atggtgtcuctgttgcttccatcgaaatc -3'	F607V
	PtPinS-F607V-R	5'- agacaccauctctgtacttgtaaccgtagt -3'	
	PtPinS-F607L-F	5'- atggtgtcuctgttgcttccatcgaaatc -3'	F607L
	PtPinS-F607L-R	5'- agagaccauctctgtacttgtaaccgtagt -3'	
	PtPinS-F607I-F	5'- atggtatcuctgttgcttccatcgaaatc -3'	F607I
	PtPinS-F607I-R	5'- agataccauctctgtacttgtaaccgtagt -3'	
P_{ERG1}	SalI-Perg1-FP	5'- gacgtcgacgtcgaatactactatgaccgc -3'	
	Perg1-HindIII-RP	5'- agaaagcttcatgacccttttctcgatatgtt -3'	
	COD71-ERG20-FP	5'- gaggaagcaacggcaggaaatatatataaacgcatgtcgatggatctgatatcaccta -3'	
	COD71-ERG20-RP	5'- tcaagaatctctctcctaatttctttttctgaagccatagcttatgacccttttctcg -3'	
	ERG20-gseq-RP	5'- ctagtcacgtggaacggtagatcg -3'	
	ERG20-gseq-FP	5'- caacaggtattggactgacataggc -3'	

Supplementary Table 8. Overview of limonene, sabinene and nerol production in engineered yeast strains under shake-flask batch cultivation conditions.

Product	Strain	Protein expressed	Product titer (mg/L)	Fold improvement	Nerol titer (mg/L)
Limonene	MIC2	Erg20p(N127W) + *Cl*LimS wt	27.97 ± 8.98	-	-
	MIC2	Erg20p(N127W) + *Cl*LimS(H570F)	32.06 ± 2.96	1.15	-
	MIC2	Erg20p(N127W) + *Cl*LimS(H570Y)	29.21 ± 9.58	1.04	-
	MIC2	Erg20p(N127W)-*Sl*NPPS1+ *Cl*LimS wt	64.64 ± 7.55	2.31	0.28 ± 0.178
	MIC2	Erg20p(N127W)-*Sl*NPPS1+ *Cl*LimS(H570F)	116.35 ± 30.26	4.16	0.03 ± 0.015
	MIC2	Erg20p(N127W)-*Sl*NPPS1+ *Cl*LimS(H570Y)	134.81 ± 21.94	4.82	0.02 ± 0.003
Sabinene	MIC2	Erg20p(N127W) + *Sp*SabS wt	17.67 ± 3.08	-	-
	MIC2	Erg20p(N127W) + *Sp*SabS(H561F)	15.97 ± 1.64	0.90	-
	MIC2	Erg20p(N127W)-*Sl*NPPS1+ *Sp*SabS wt	25.44 ± 10.34	1.44	1.01 ± 0.147
	MIC2	Erg20p(N127W)-*Sl*NPPS1+ *Sp*SabS(H561F)	72.39 ± 17.55	4.10	0.07 ± 0.020

Fold improvement corresponds to titer improvement compared to the corresponding Erg20p(N127W) and wild-type terpene synthase expressing MIC2 strain. Errors correspond to the mean absolute deviation (MAD) around the mean (*n* = 3 different yeast transformations processed and analyzed separately). Source data are provided as a Source Data file.

SUPPLEMENTARY FIGURES

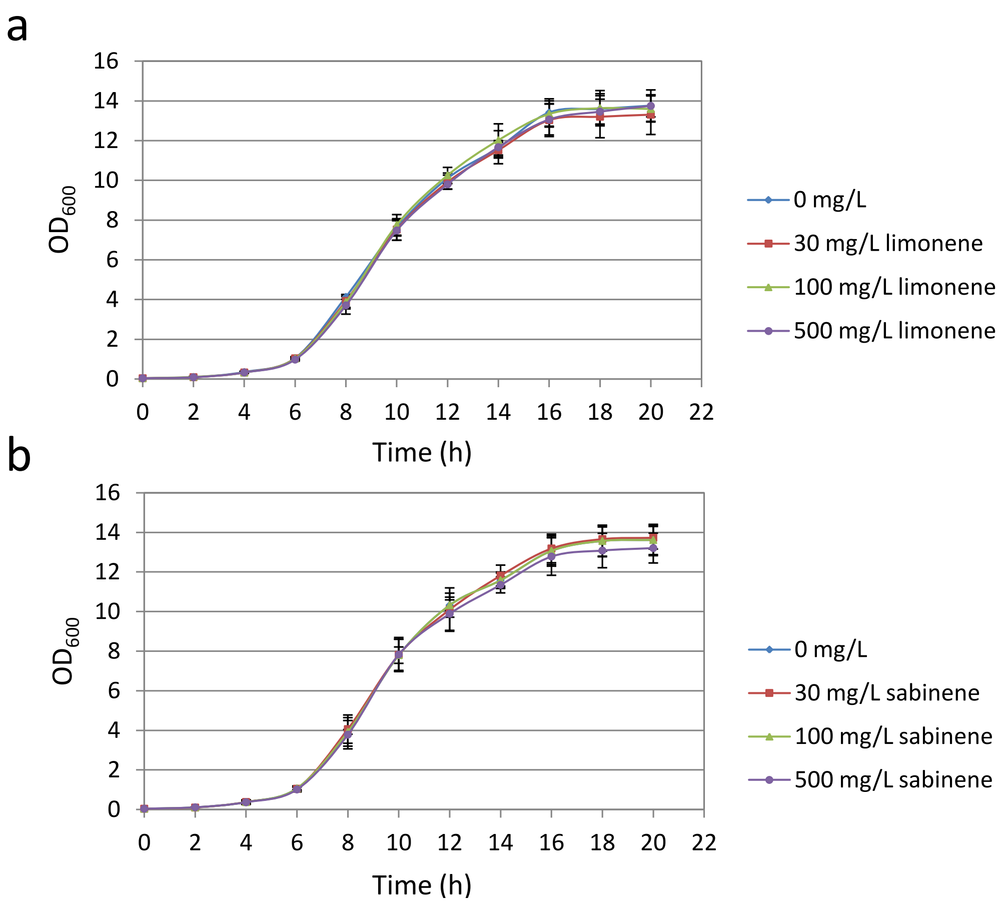

Supplementary Figure 1. Evaluation of the effect of monoterpenes in the growth of AM94 yeast cells. To examine whether the low monoterpene production titers observed in yeast are due to product toxicity, we evaluated the growth of AM94 yeast cells under conditions that recapitulate production of 0 mg/L (blue), 30 mg/L (red), 100 mg/L (green) and 500 mg/L (violet) of limonene (**a**) or sabinene (**b**). To achieve this, yeast cells were overlaid with 0.1 volume of dodecane containing 0 (blue), 300 (red), 1000 (green) and 5000 mg/L (violet) of limonene or sabinene. In all conditions tested, we did not observe any significant toxicity of the two monoterpenes. Errors correspond to the mean absolute deviation (MAD) around the mean (n = 3 different starting cultures for each condition). Source data are provided as a Source Data file.

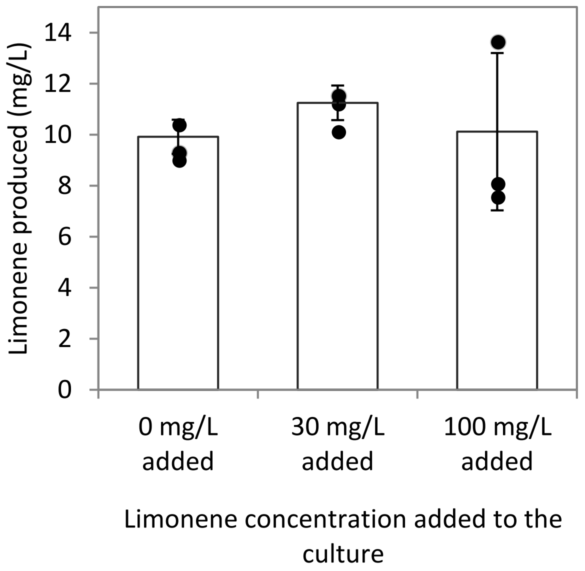

Supplementary Figure 2. The presence of the monoterpene product does not inhibit further production of limonene by the yeast cells. To evaluate whether monoterpene productivity was limited by the presence of the product, we evaluate the production of limonene by *Cl*LimS in AM94 cell cultures supplemented with different concentrations of limonene (by adding 0 mg/L, 300 mg/L and 1 g/L in the dodecane overlay so as to correspond to the production of 0 mg/L, 30 mg/L and 100 mg/L limonene by the culture). In all cases, the concentration of limonene in the overlay increased by a similar amount (y-axes), suggesting that limonene productivity by the yeast cells was similar in all conditions and no inhibition was observed. Errors correspond to the mean absolute deviation (MAD) around the mean. (*n* = 3 independent yeast transformations were used as separate seed cultures, which were then each treated with the three limonene concentrations. Source data are provided as a Source Data file.

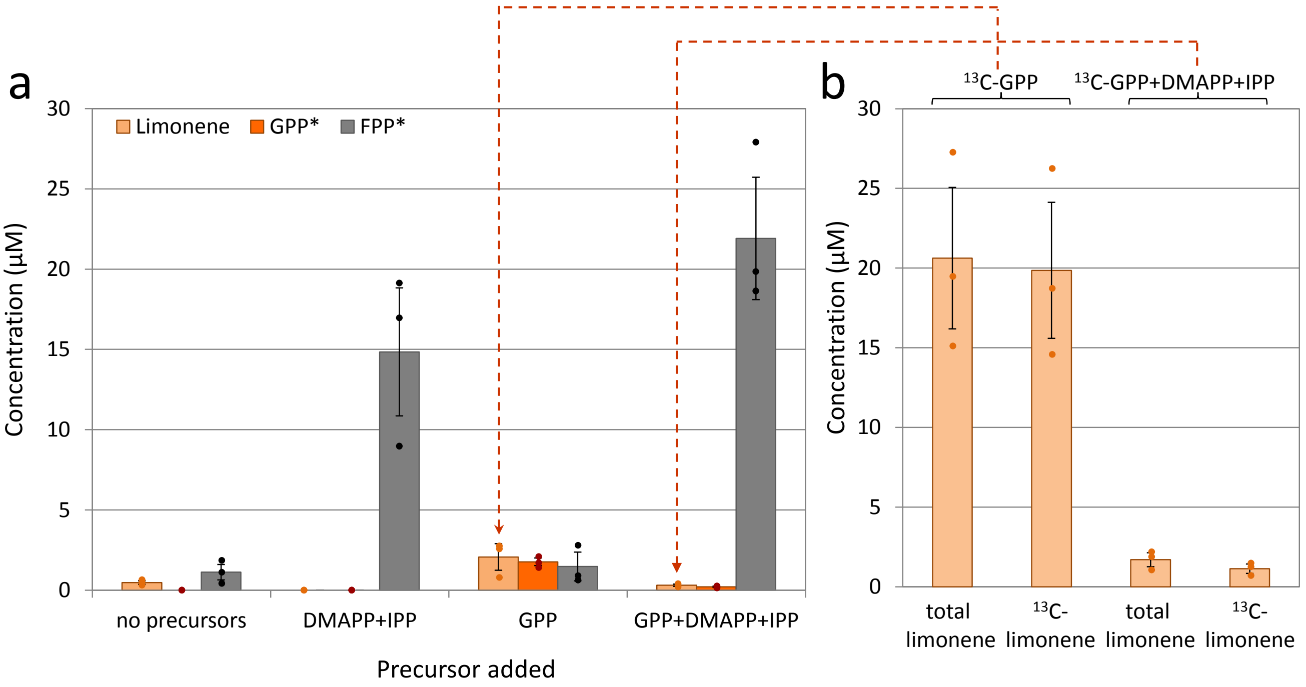

Supplementary Figure 3. Evaluation of substrate flux. a. AM94 yeast cells expressing *Cl*LimS and Erg20p were disrupted with glass beads, the cell extract was supplemented with specific amounts of different isoprenoid precursors, and the formation of limonene, GPP and FPP was measured by GC-FID analysis. Where indicated, the precursors were added at the following concentrations: "DMAPP+IPP" = 25 µM DMAPP and 100 µM IPP, "GPP" = 25 µM GPP, and "GPP+DMAPP+IPP" = 25 µM DMAPP, 100 µM IPP and 25 µM GPP. GPP* and FPP* denote the estimated GPP and FPP concentration calculated following acid hydrolysis of the reactions (linalool produced by hydrolysis of GPP (showed as GPP*) and nerolidol produced by hydrolysis of FPP (showed as FPP*)). As shown by the increase in FPP*, in the presence of DMAPP and IPP, most of the precursors were used by the yeast extracts to synthesize FPP, and only a small amount of the GPP added was converted to limonene by *Cl*LimS. Errors correspond to the mean absolute deviation (MAD) around the mean. (*n* = 3 technical replicates). **b.** The experiment described above was performed using ^{13}C-GPP instead of unlabeled GPP to evaluate the amount of limonene produced by the added GPP. Reactions were extracted with hexane and analyzed by GC-APCI-QqToF. Mass spectra of the labelled and unlabeled limonene produced are provided in Supplementary Fig. 11a. Although in the absence of DMAPP and IPP, most of limonene produced was derived from the labelled substrate (indicating that the *Cl*LimS was active and able to convert the substrate efficiently), less than 10% of the ^{13}C-GPP was converted to limonene in the presence of DMAPP and IPP because most of it was channeled to FPP. Errors correspond to the mean absolute deviation (MAD) around the mean (*n* = 3 technical replicates). Source data are provided as a Source Data file.

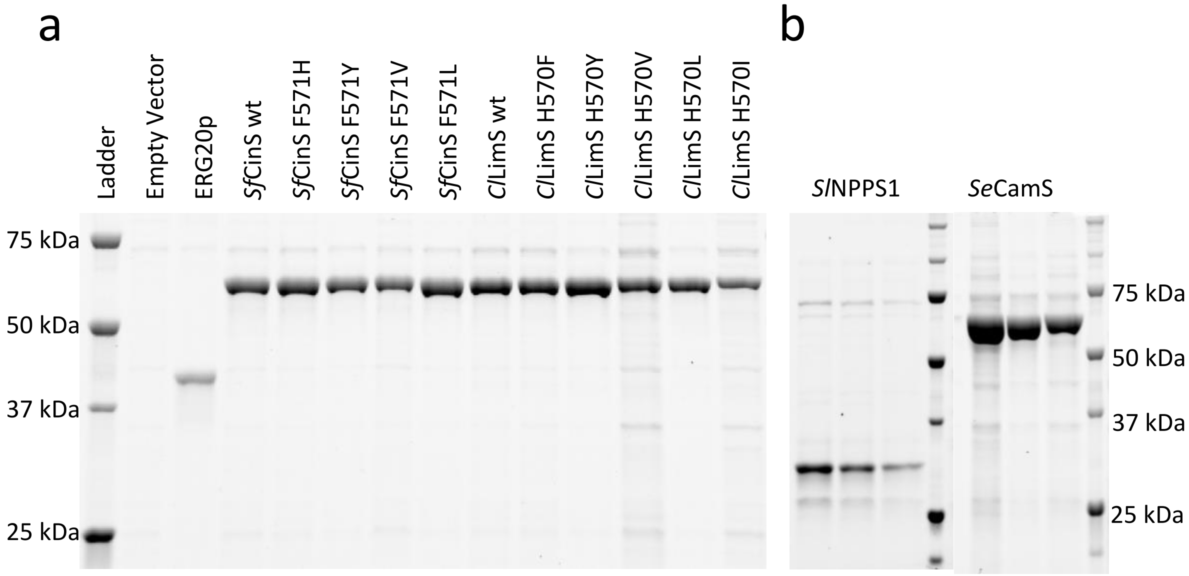

Supplementary Figure 4. SDS-PAGE of the recombinant proteins purified from *E. coli* and used in the *in vitro* assays. All proteins, N-terminally fused to a 6xHis tag, were purified using Ni^{2+}-affinity chromatography. Cells transformed with the empty expression vector were subjected to the same purification protocol and the elutions were used as negative control in the *in vitro* assays. **a**. SDS-PAGE of the empty vector, Erg20p (40kDa), *Sf*CinS1 wild-type and mutants (69 kDa), and *Cl*LimS wild-type and mutants (70kDa). **b.** SDS-PAGE gel of purified recombinant His-tagged *Sl*NPPS1 (34 kDa) and *Se*CamS (70 kDa). Source data are provided as a Source Data file.

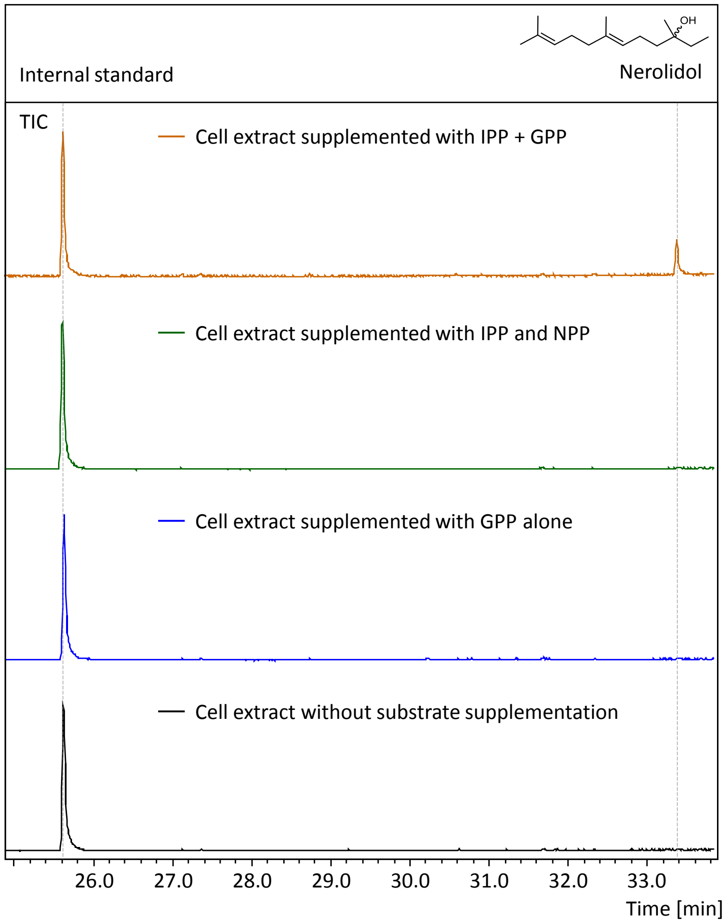

Supplementary Figure 5. Evaluation of the ability of yeast cells to use NPP as a substrate for the production of larger diphosphates. Chromatograms showing the acid hydrolysis products of Erg20p expressing AM94 yeast cell extract incubated with 120 µM IPP and 40 µM GPP (brown), 120 µM IPP and 40 µM NPP (green) or with 40 µM GPP (blue). Acid hydrolysis of the product of Erg20p with IPP and GPP produces nerolidol, which is the main hydrolysis product of FPP. Nerolidol was confirmed to originate from GPP by isotope labelling of GPP, as shown in Supplementary Figure 11b. Erg20p was not able to use NPP as substrate to synthesize larger diphosphate molecules. *Trans-β*-caryophyllene was used as internal standard. Source data are provided as a Source Data file.

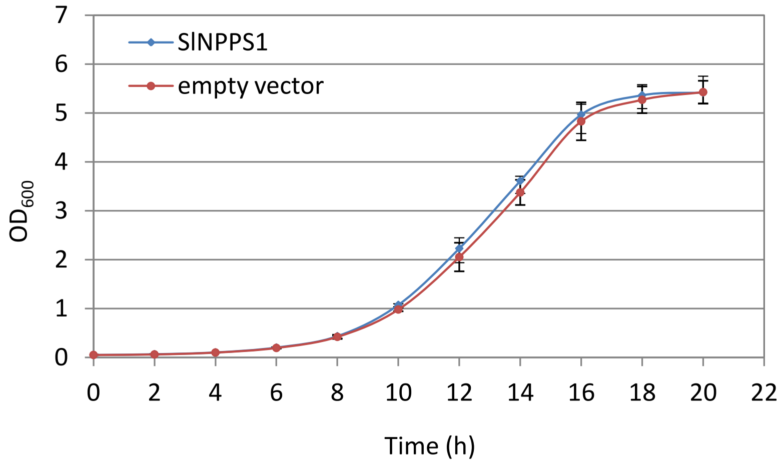

Supplementary Figure 6. Growth curves of yeast cells overexpressing *Sl*NPPS1 or containing the empty vector. We observed no growth retardation caused by expression of *Sl*NPPS1. Errors correspond to the mean absolute deviation (MAD) around the mean (n = 3 different yeast transformations used as starting cultures). Source data are provided as a Source Data file.

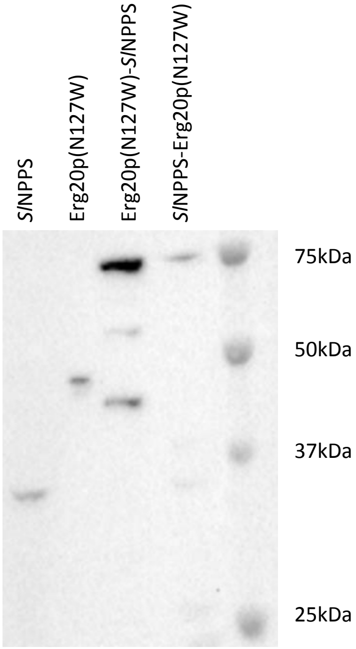

Supplementary Figure 7. Western blot analysis of myc-tagged *Sl*NPPS1, Erg20p(N127W) and two fusion proteins in different orientation, Erg20p(N127W)-*Sl*NPPS1 and *Sl*NPPS1-Erg20p(N127W) fusion. The level of *Sl*NPPS and Erg20p(N127W) proteins and their fusions were evaluated using Western blotting and probing against the N-terminal myc epitope. Erg20p(N127W)-*Sl*NPPS1 achieves higher protein levels than each of the other three forms. Source data are provided as a Source Data file.

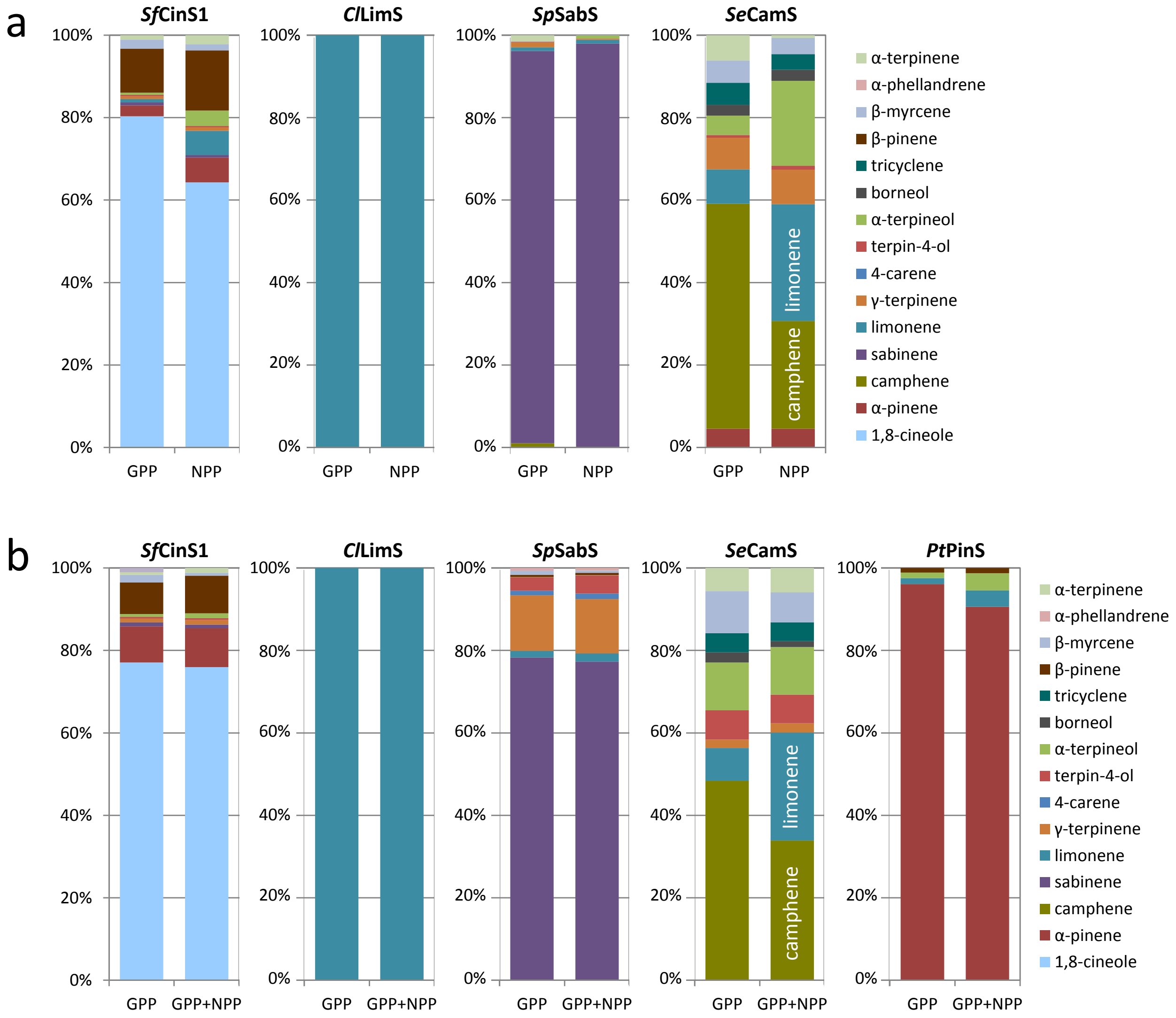

Supplementary Figure 8. In vitro and in vivo product profiles of the canonical terpene synthases. a. The product profile of purified enzyme in *in vitro* reactions using either GPP or NPP as substrates. **b.** Profile of monoterpenes produced the selected monoterpene synthases when expressed in yeast cells producing GPP only or GPP together with NPP. The minor differences in the product profile obtained *in vitro* versus *in vivo* in the case of *Sf*CinS1 and *Sp*SabS is likely due to differences in the performance of these enzymes in different environments and has been observed previously with other terpene synthases[4]. Samples were prepared independently from 3 different yeast transformations, processed and analyzed separately (n=3 biologically independent samples). The average yield of each compound between the samples was calculated and the percentage of each compound in the product profile was deduced. Compounds identified by comparison to authentic standards where possible, otherwise by comparison to reported enzyme profiles in combination with published Kovats indices and mass-spectra. Source data are provided as a Source Data file.

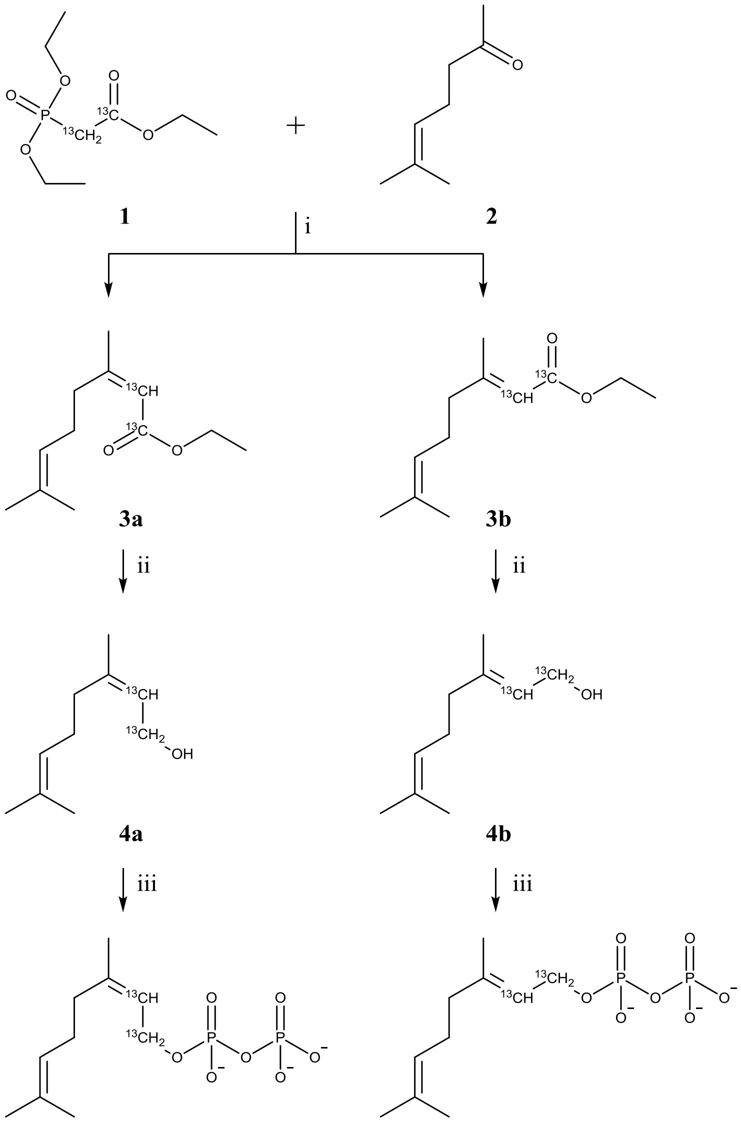

5a 5b

Supplementary Figure 9. Chemical synthesis of (Z)- [1,2- $^{13}C_2$]-neryl pyrophosphate 5a and (E)- [1,2- $^{13}C_2$]-geranyl pyrophosphate 5b. (Z)- [1,2- $^{13}C_2$]-neryl pyrophosphate **5a** and its isomer (E)- [1,2- $^{13}C_2$]-geranyl pyrophosphate **5b** were chemically synthesized as recently described for the synthesis of (E)-2-methylgeranyl pyrophosphate[6] and as shown in the schema. Reagents and reaction conditions: i) LiCl, DBU, solvent free, rt, 24 h, 90 %. ii) DIBAL-H, Et_2O, -78 °C, 1h, quant. iii) $(Ph)_3P/CBr_4$, $[(n\text{-}Bu)_4N]_3HP_2O_7$, Dry CH_3CN, rt, 24h.

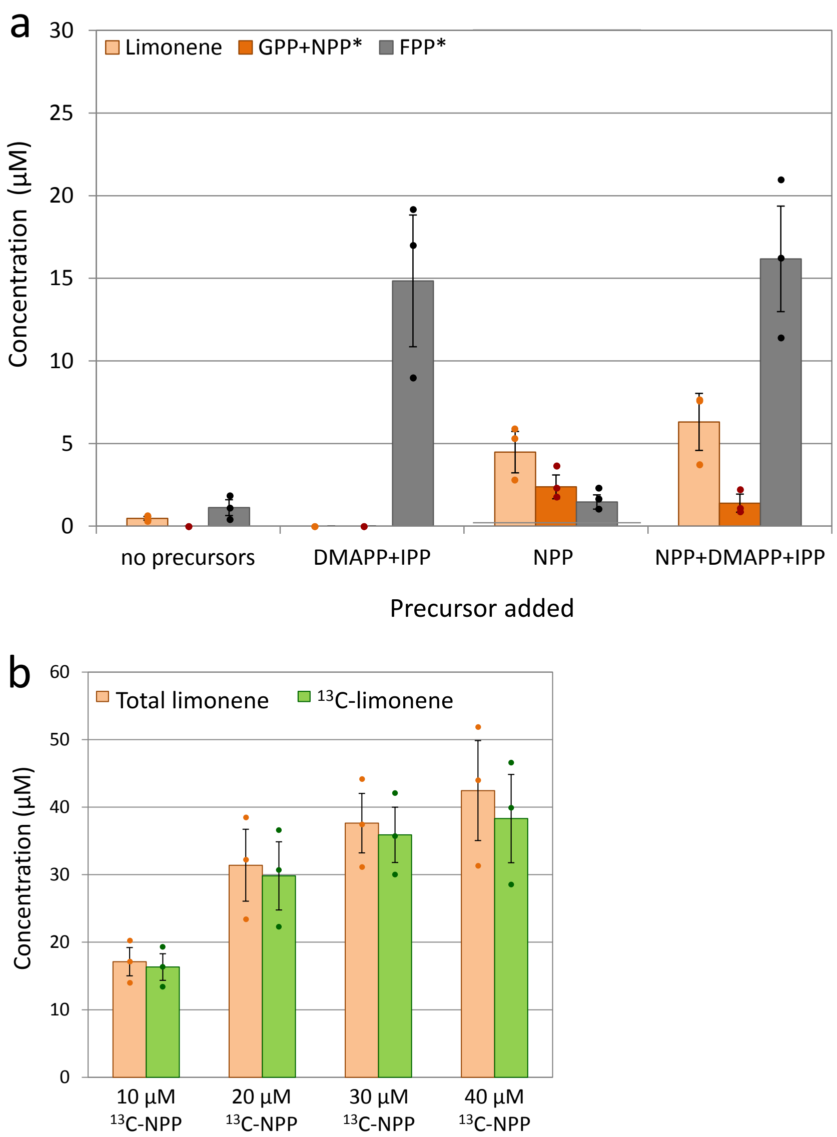

Supplementary Figure 10. NPP is used exclusively for the production of monoterpenes and not consumed for the synthesis of larger prenyl diphosphates. a. Extracts of AM94 yeast cells expressing *Cl*LimS and Erg20p were supplemented with DMAPP, IPP and NPP precursors. After 1 h incubation at 30 °C, the samples were treated by acid hydrolysis and the resulted products were measured by GC-FID. In addition to limonene synthesized by *Cl*LimS, we estimated the concentration of GPP and NPP, and FPP by measuring their respective hydrolysis products (linalool for NPP and GPP, denoted as "NPP+GPP*"); and nerolidol (for FPP, shown as "FPP*"). Contrary to what was observed in the case of GPP (Supplementary Figure 1), NPP is not consumed for the synthesis of larger prenyl diphosphates but can be utilized efficiently for limonene synthesis. **b.** We supplemented cells with labelled ^{13}C-NPP to confirm that the limonene produced under the conditions tested in panel A was mostly produced by NPP. Extracts were supplemented with 40 µM DMAPP, 120 µM IPP and increased concentrations of ^{13}C-NPP, directly extracted with hexane, and analyzed by GC-APCI-QqToF. To trace the flux of labeled NPP in the produced limonene, we examined the 2 units shift in the resulted mass spectra (Supplementary Figure 11a). Total limonene (orange) and limonene produced from ^{13}C-NPP (green) is shown. The majority of the limonene detected is produced by ^{13}C-NPP, despite the presence of DMAPP and IPP precursors, which are channeled by Erg20p towards the synthesis of FPP. Errors correspond to the mean absolute deviation (MAD) around the mean (n = 3 technical replicates). Source data are provided as a Source Data file.

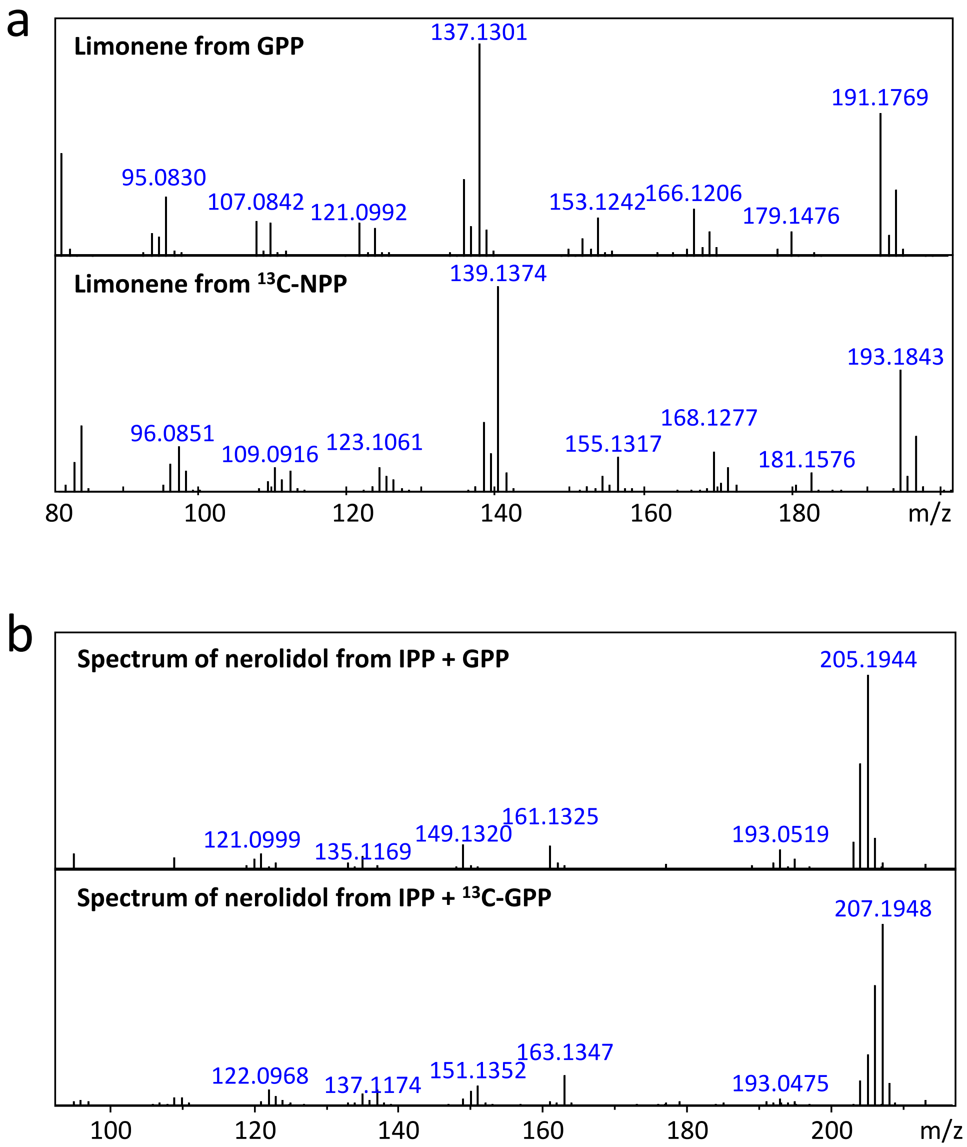

Supplementary Figure 11. Mass spectra of the products synthesized in enzymatic reactions of yeast extracts containing *Cl*LimS with labeled and unlabeled substrates. a. The mass spectrum of limonene produced from GPP or ^{13}C-NPP is shown. ^{13}C-NPP derived limonene exhibits a shift of the *m/z ratio* by 2 units. **b.** Mass-spectrum of nerolidol produced by yeast extracts supplemented with IPP and GPP (top) or IPP and ^{13}C-GPP (bottom). A shift of 2 units is observed in the *m/z* ions of nerolidol produced from ^{13}C-GPP, confirming that this substrate is channeled towards the synthesis of larger diphosphates. Spectra obtained using GC-APCI-QqToF analysis.

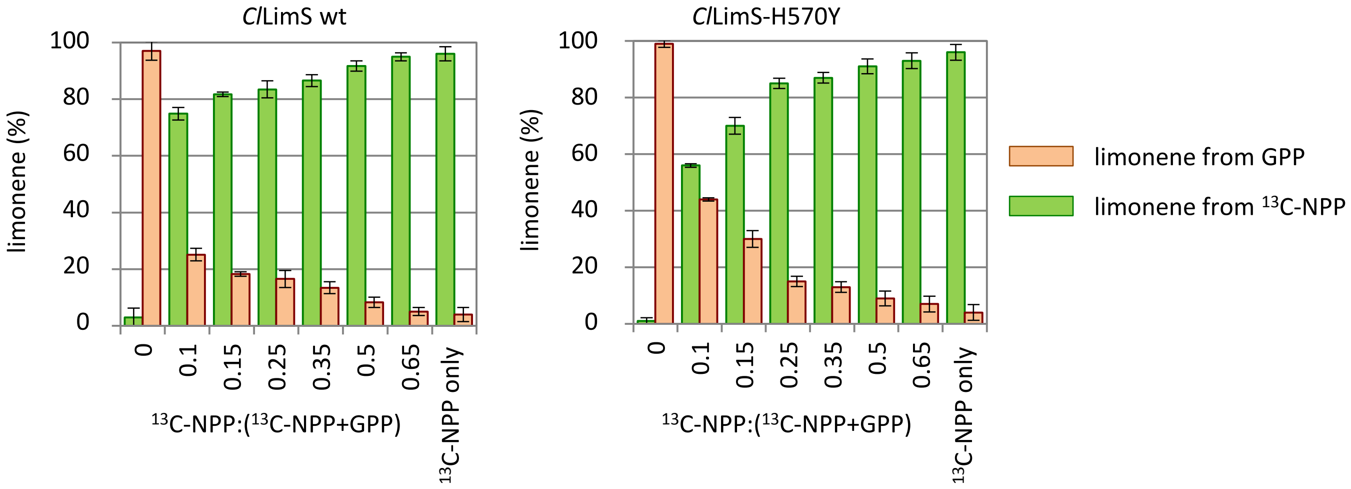

Supplementary Figure 12. Substrate competition experiment. We evaluated the relative efficiency of cell extracts of yeast cells expressing *Cl*LimS wt (left) and *Cl*LimS(H570Y) (right) to synthesized limonene from the two isomeric substrates, GPP or NPP, using isotopically labelled substrate to differentiate between the limonene produced by each of the substrates. In this competition experiment, a range of different ratios of ^{13}C-NPP:(^{13}C-NPP+GPP) were evaluated in a fixed concentration of DMAPP and IPP (detailed in Data Source). Incorporation of the ^{13}C-NPP or GPP in limonene was monitored by the difference in the mass spectrum containing *m/z* ions larger by 2 units, recorded using GC-APCI-QqToF analysis. This experiment corroborates the previous findings of full incorporation of the NPP substrate in the product formed, while only reduced usage of the GPP precursor, which is consumed by endogenous Erg20p. At a ratio as low as 0.1 ^{13}C-NPP:(^{13}C-NPP+GPP), the amount of limonene produced from ^{13}C-NPP surpasses that synthesized from GPP. Using the highly NPP-specific *Cl*LimS(H570Y) variant, more than 2-fold increase in the amount of ^{13}C-NPP-derived limonene was obtained as previously observed from the yeast product profile observed. Error bars correspond to weighted standard error between 4 calculated ^{13}C-NPP:(^{13}C-NPP+GPP) ratios as described in the Source Data file under Supplementary Figure 12 support. Source data are provided as a Source Data file.

```
SfCinS1  (552) VSANLARMAQWIYQHESDGFGMQHSLVNKMLRDLLFHRYE----------
 ClLimS  (552) FLLNLVRMSHFMYLHG-DGHGVQNQETIDVGFTLLFQPIPLEDKHMAFTA
 SeCamS  (540) CGMNIARTGQTIYQHG-DGHGIQNYEIQNRIYKLFFDPIAVSIP------
 PtPinS  (589) VAFDICRVFHYGYKYR-DGFSVASIEIKNLVTRTVVETVPL---------
 SpSabS  (543) VATNLARAAQFIYLDG-DGHGVQHSEIHQQMGGLLFQPYV----------
```

Supplementary Figure 13. Alignment of the plant terpene synthases used in this study showing the residues corresponding to F571 in *Sf*CinS1. A conserved DG pair (marked in red) is located upstream of F571. This residue is a phenylalanine in *Sf*CinS1 and *Pt*PinS, or a histidine in *Cl*LimS, *Sp*SabS and *Se*CamS (highlighted in yellow).

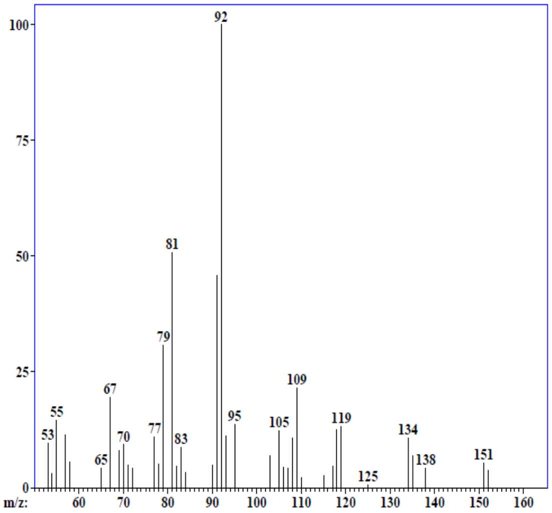

Supplementary Figure 14. Mass spectrum of the *trans*-sabin-3-ol product obtained in yeast cells expressing *Sl*NPPS1, *Sp*SabS(H561F) and CYP750B1. The mass spectrum of the hydroxylated compound produced by CYP750B1 enzyme in sabinene-producing yeast cells match the mass spectrum of *trans*-sabin-3-ol reported by Gesell and co-workers[5].

REFERENCES

1 Ignea, C. *et al.* Positive genetic interactors of HMG2 identify a new set of genetic perturbations for improving sesquiterpene production in Saccharomyces cerevisiae. *Microb. Cell Fact.* **11**, 162 (2012).

2 Ignea, C. *et al.* Efficient diterpene production in yeast by engineering Erg20p into a geranylgeranyl diphosphate synthase. *Metab. Eng.* **27**, 65-75 (2015).

3. Ignea, C., Pontini, M., Maffei, M. E., Makris, A. M. & Kampranis, S. C. Engineering monoterpene production in yeast using a synthetic dominant negative geranyl diphosphate synthase. *ACS Synth. Biol.* **3**, 298-306 (2014).

4 Yoshikuni, Y., Martin, V. J., Ferrin, T. E., & Keasling, J. D. Engineering cotton (+)-delta-cadinene synthase to an altered function: germacrene D-4-ol synthase. *Chem. Biol* **13**, 91-8 (2006).

5. Gesell, A. et al. The gymnosperm cytochrome P450 CYP750B1 catalyzes stereospecific monoterpene hydroxylation of (+)-sabinene in thujone biosynthesis in western redcedar. *Plant Physiol*. 168, 94-106 (2015).

6. Ignea, C. *et al.* Synthesis of 11-carbon terpenoids in yeast using protein and metabolic engineering. *Nat. Chem. Biol.* **14**, 1090-1098 (2018).
