# Supplementary material for: Orthogonal monoterpenoid biosynthesis in yeast constructed on an isomeric substrate
Source: Nat Commun. 2019 Aug 23;10:3799. doi: 10.1038/s41467-019-11290-x (PMC6707142; doi:10.1038/s41467-019-11290-x)
Supplement: Supplementary file 3 — Reporting Summary [file 41467_2019_11290_MOESM3_ESM.pdf]

nature research

Corresponding author(s): Sotirios C. Kampranis

Last updated by author(s): Jun 30, 2019

Reporting Summary

Nature Research wishes to improve the reproducibility of the work that we publish. This form provides structure for consistency and transparency in reporting. For further information on Nature Research policies, see Authors & Referees and the Editorial Policy Checklist.

Statistics

For all statistical analyses, confirm that the following items are present in the figure legend, table legend, main text, or Methods section.

n/a	Confirmed	
☐	☒	The exact sample size (*n*) for each experimental group/condition, given as a discrete number and unit of measurement
☐	☒	A statement on whether measurements were taken from distinct samples or whether the same sample was measured repeatedly
☒	☐	The statistical test(s) used AND whether they are one- or two-sided<br>*Only common tests should be described solely by name; describe more complex techniques in the Methods section.*
☒	☐	A description of all covariates tested
☒	☐	A description of any assumptions or corrections, such as tests of normality and adjustment for multiple comparisons
☐	☒	A full description of the statistical parameters including central tendency (e.g. means) or other basic estimates (e.g. regression coefficient) AND variation (e.g. standard deviation) or associated estimates of uncertainty (e.g. confidence intervals)
☒	☐	For null hypothesis testing, the test statistic (e.g. *F*, *t*, *r*) with confidence intervals, effect sizes, degrees of freedom and *P* value noted<br>*Give P values as exact values whenever suitable.*
☒	☐	For Bayesian analysis, information on the choice of priors and Markov chain Monte Carlo settings
☒	☐	For hierarchical and complex designs, identification of the appropriate level for tests and full reporting of outcomes
☒	☐	Estimates of effect sizes (e.g. Cohen's *d*, Pearson's *r*), indicating how they were calculated

Our web collection on statistics for biologists contains articles on many of the points above.

Software and code

Policy information about availability of computer code

Data collection	BRUKER DALTONICS MS WORKSTATION V8.2.1 for GC-TQ MS quantification,<br>and GCMS Solution for Shimadzu GC-MS quantification.
Data analysis	AMDIS GC/MS Analysis Version 2.70 for mass spectra analysis<br>UCSF Chimera for structural analysis.<br>ChemDraw Professional 15.1 for chemical structures, MS Excel for bar charts and graphs, and MS Powerpoint for the preparation of illustrations. WinCurveFit for curve fitting of kinetic analysis data.

For manuscripts utilizing custom algorithms or software that are central to the research but not yet described in published literature, software must be made available to editors/reviewers. We strongly encourage code deposition in a community repository (e.g. GitHub). See the Nature Research guidelines for submitting code & software for further information.

Data

Policy information about availability of data

All manuscripts must include a data availability statement. This statement should provide the following information, where applicable:

- Accession codes, unique identifiers, or web links for publicly available datasets
- A list of figures that have associated raw data
- A description of any restrictions on data availability

Data supporting the findings of this work are available within the paper and its Supplementary Information files. A reporting summary for this Article is available as a Supplementary Information file. The datasets generated and analyzed during the current study are available from the corresponding author upon request. The source data underlying Tables 1, 2 and 3; Figures 2c, 2d, 3a, 3b, 3c, 3d, 3e, 4a, 5a, 5b, 5c, 5d, and 5g; Supplementary Tables 2, 3, 4, 5, 6, 8; Supplementary Figures 1, 2, 3a, 3b, 4, 6, 7, 8a, 8b, 10a, 10b, 12a, and 12b are provided as a Source Data file.

Field-specific reporting

Please select the one below that is the best fit for your research. If you are not sure, read the appropriate sections before making your selection.

☒ Life sciences ☐ Behavioural & social sciences ☐ Ecological, evolutionary & environmental sciences

For a reference copy of the document with all sections, see nature.com/documents/nr-reporting-summary-flat.pdf

Life sciences study design

All studies must disclose on these points even when the disclosure is negative.

Sample size	Not relevant to this study. Please refer to point 3, replication, for how samples were allocated and analyzed.
Data exclusions	No data exclusion.
Replication	All determinations were repeated in triplicates. All attempts at replication of the experiments using re-establishing of yeast strain and reanalysis of the products have been successful.
Randomization	20-30 yeast colonies was randomly selected for the analysis of each of the constructed yeast strains.
Blinding	GC-MS analysis of the products was carried out by blind labeling of the samples and automated analysis

Reporting for specific materials, systems and methods

We require information from authors about some types of materials, experimental systems and methods used in many studies. Here, indicate whether each material, system or method listed is relevant to your study. If you are not sure if a list item applies to your research, read the appropriate section before selecting a response.

Materials & experimental systems

n/a	Involved in the study	
☒	☐	Antibodies
☒	☐	Eukaryotic cell lines
☒	☐	Palaeontology
☒	☐	Animals and other organisms
☒	☐	Human research participants
☒	☐	Clinical data

Methods

n/a	Involved in the study	
☒	☐	ChIP-seq
☒	☐	Flow cytometry
☒	☐	MRI-based neuroimaging
