# Supplementary material for: Orthogonal monoterpenoid biosynthesis in yeast constructed on an isomeric substrate
Source: Nat Commun. 2019 Aug 23;10:3799. doi: 10.1038/s41467-019-11290-x (PMC6707142; doi:10.1038/s41467-019-11290-x)
Supplement: Supplementary file 2 — Peer Review [file 41467_2019_11290_MOESM2_ESM.pdf]

Reviewers' comments:

Reviewer #1 (Remarks to the Author):

The present work by Ignea et al. describes the engineering of canonical terpene synthases in yeast. In contrast to GPP, the cis isomer neryl diphosphate (NPP) is used in vivo as an alternative substrate. It was shown that the ligand is stable in yeast. NPP-specific monoterpene synthases were co-expressed, yielding the respective products. Subsequently the efficiency and specificity for NPP-processing was optimized. The authors performed an elaborate mutagenesis on cineole synthase 1. In this individual study, modification of Phe571 was identified to have a preference for either GPP or NPP. Taken together, the presented results might be of some interest for experts and non-experts in the field of biochemistry / biotechnology.

Major revisions are requested:
1) Orthogonal monoterpenoid biosynthesis has already been described in bacteria. Ignea et al. argue that compared to yeast, the prokaryotic system lacks characteristic P450 enzymes and monoterpenoids exhibit significant toxicity to bacteria. Examples have to be included in the revised version, otherwise the introduced eukaryotic system lacks novelty.

2) Enzyme kinetics [K(m) and k(cat)] are of significant importance in the presented (biotechnological) study – values and applied methods have to be included in detail into the main text.

3) The synthetic metabolism should be confirmed at least in one case by isotope labelling experiments.

Minor comments:
1) Figure 4b seems to be incomplete.

2) Even though five different enzymes have been described to accept the alternate substrate, detailed analysis was performed on SfCinS1 – please write a short paragraph why this enzyme was selected.

3) In the abstract, the results and the performed approach appear exaggerated.

Reviewer #2 (Remarks to the Author):

General Comments
In this paper authors have identified a synthetic orthogonal pathway for the production of neryl diphosphate that serves as scaffold for the terpene production including limonene, pinene etc. They first ensure that the new enzyme that condenses the DMAPP and IPP is truly orthogonal and then they express monoterpene synthases that have activity towards NPP when they discover nerol (a hydrolysis product of NPP) suggesting that the enzymes for orthogonal pathway are not optimal. They subsequently engineer these synthases for higher activity on NPP and show that the synthetic pathways produce up to 7-18 times more product than the native pathways. They have exploited the concept of orthogonality and shown that orthogonal pathways can lead to higher production however they have not done the dynamic regulation that can truly exploit these synthetic pathways.

Major Comments

The introduction to the paper is very brief. For example, while the concept of orthogonality has been proposed before, it has not yet prevalent in metabolic engineering. Hence, authors could include more details introducing the concept and how it connects to dynamic regulation (as this is not so obvious at least the way it is written) and how it might different from the orthogonality common in synthetic

biology (e.g. noncanonical AAs, orthogonal genetic parts and circuits).

While the authors have shown that the 5 monoterpene synthases have activity with both NPP and GPP, this reviewer feels it is important to distinguish the extent of production through the orthogonal pathway vs the native pathway for the original as well as the native enzymes.

The manuscript ends a bit abruptly given that the paper is leading towards the dynamic regulation and one would have really liked to see some results on that part but I do understand scope limitations.

Minor Comments

For the Figure 4A and the enzyme SfCin did the authors consider mutations at both N338 and F571. These days such mutants can be easily ordered...

Figure 1 can be improved to indicate the different products that the authors are considering as well as the terpene synthase enzymes that were being engineered in order to help with the flow of the story.

Reviewer #3 (Remarks to the Author):

In the manuscript from Ignea et al., "Orthogonal monoterpenoid biosynthesis in yeast constructed on an isomeric substrate," the authors introduce a neryl pyrophosphate synthase into the cell, and demonstrate that NPP-specific monoterpene synthases can convert NPP into monoterpene products. They call this pathway "orthogonal" to yeast metabolism, because it has only a single branchpoint from central metabolism. The authors show that strains carrying the NPP-specific monoterpene synthases accumulate of NPP, suggesting a kinetic limitation. To address this, the authors perform protein engineering on canonical monoterpene synthases to enable the use of NPP as a substrate, identifying several key residues in the synthases that determine substrate specificity. The authors then observe high titers of monoterpenes when using these engineered synthase on concert with the NPP producing enzymes.

There is some creative metabolic engineering in this work to improve product titers, and there is some thorough protein engineering work to alter the substrate specificity of the monoterpene synthases. However, these results do not support the larger claims of the work around the greater efficacy of an "orthogonal" pathway relative to a native pathway. Because of this, I do not see this work as suitable for Nature communications.

To me it seems that the framing of the work is is distracting rather than clarifying, and does the work a disservice. In trying to demonstrate novelty, the authors make claims that are based in opinion rather than evidence.

For example, a central claim of the work is that producing monoterpenes in yeast is difficult because cells are not optimized to produce GPP - whereas producing sesquiterpenes has been successful at scale because yeast cells are optimized for the production of FPP-derived molecules. This doesn't make much sense to me, as cells must produce stoichiometrically equivalent amounts of GPP as FPP, given that GPP is a direct precursor to FPP. Cells are no more optimized to produce FPP than they are optimized to produce GPP. There are far simpler explanations for the observation that monoterpenes have not been as effectively produced in S. cerevisiae - for instance, monoterpenes are generally more toxic to cells than sesquiterpenes.

The authors also claim that production of monoterpenes in E. coli is higher than in S. cerevisiae because there are no major biomass generating pathways that depend on GPP. This is an important prediction of their "orthogonality" claim, but there is no actual evidence that the lack of competing pathways in E. coli has anything to do with the higher titers. In fact, it would logically follow from this

claim that production of sesquiterpenes should also be more efficient in E. coli, which is clearly not the case.

The idea that the NPP synthase offers more "orthogonality" than simply introducing a monoterpene synthase is also a subjective one. It could just as easily be claimed that the NPP synthase-using pathway is a two-step heterologous pathway to produce monoterpenes drawing from IPP/DMAPP, whereas the "canonical" method to product monoterpenes is a one-step pathway drawing from GPP. The idea of "orthogonality" seems semantic, rather than directly relevant to their results. The higher levels of production in the presence of the NPP synthases could be explained by enzyme kinetics as much as pathway structure - this simpler explanation needs to be ruled out before claims of orthogonality could be made.

There are also some very basic experimental issues with the paper. For example, in many of the figures, "samples were analyzed in triplicates." To me this sounds like technical replicates -- am I misunderstanding this? Biological replicates are necessary to understand the reproducibility of these results - technical replicates are not the the appropriate test for establishing reproducibility. The language makes me think that the authors performed only technical replicates for many of the experiments.

I am also very puzzled by the lack of information around the last claim of the work, which is that the NPP-specific synthases enabled production of monoterpenes with higher efficiency than the native pathway. Given that record monoterpene titers are a primary claim in the abstract, these results need to be in a figure, properly described, with described methods, appropriate statistical analysis and genotypic description of strains. I could not tell from the manuscript how the authors achieved these titers of 130 mg/L and 72.7 mg/L, and I do not see any genotype information for these strains. The description of their control, "the same strain by the native pathway," is very vague. What is meant by the "native pathway" here? Does it include any of the Erg20 engineering shown in Figure 2? None of this information is provided. Moreover, it seems like the logical experiment would be to put the newly engineered NPP-specific synthases into the strains constructed for Figure 2, and determine whether there is a large increase in monoterpene levels, and a decrease in NPP levels.

Subject: Response to reviewers comments (NCOMMS-18-29430)

We would like to sincerely thank the reviewers for their thorough and constructive comments. We have addressed all major concerns with additional experimental evidence that includes experiments with isotopically labeled precursors, kinetic evaluation, and additional mutagenesis studies. Furthermore, we expanded the scope of the manuscript by establishing dynamic regulation of the orthogonal pathway, leading to further productivity improvements. In the revised manuscript, we are also providing a clearer description of the concept of orthogonality and its application in pathway design, which was not obvious in the initial manuscript leading to misunderstanding of the findings and the novelty of the approach. Please find below our point-by-point response:

Reviewer #1:

Comment 1.1: Orthogonal monoterpenoid biosynthesis has already been described in bacteria. Ignea et al. argue that compared to yeast, the prokaryotic system lacks characteristic P450 enzymes and monoterpenoids exhibit significant toxicity to bacteria. Examples have to be included in the revised version, otherwise the introduced eukaryotic system lacks novelty.

Response: Following the reviewer's suggestion, we have now expanded the discussion on the toxicity of monoterpenes in **lines 83-87 and lines 372-375** and we have included several additional references to further substantiate this claim (toxic in bacteria: Dunlop, *et al.*, 2011; George *et al.*, 2018; Todorovic *et al.*, 2016; Jongedijk *et al.*, 2016 – Not toxic in yeast: Denby *et al.*, 2018; Brennan *et al.*, 2012).

However, we would like to take the opportunity here to clarify a possible misunderstanding of the application of the concept of orthogonality in the case of metabolic pathways. Reading the present comment of reviewer #1, and taking in to consideration comment 2.1 of reviewer #2, we understand that our description of orthogonality in the initial manuscript may not have been sufficiently clear to explain the approach described in this work. While the term "heterologous pathway" describes any foreign or synthetic pathway introduced in an organism, an "orthogonal pathway" is a heterologous pathway that has only one junction point with the cell's native metabolism, facilitating the uncoupling of the heterologous pathway from biomass generating pathways (Pandit et al., 2017, de Lorenzo, 2011). We have now included a clearer description of the term in the introduction in **lines 39-49**.

In light of this clarification, and in response to the concern expressed by reviewer #1 regarding the novelty of the approach, we would like to explain that although heterologous monoterpenoid biosynthesis has indeed previously been introduced in bacteria (and yeast), an orthogonal pathway has not. This is because bacteria already synthesize farnesyl diphosphate (FPP), which is essential for growth, and therefore any heterologous monoterpenoid pathway based on GPP is not orthogonal to the

host because it feeds back to FPP synthesis (Mendez-Perez *et al.*, 2017). The orthogonal, NPP-based, pathway proposed here will also be orthogonal when introduced in bacteria. The novelty of our work is exactly at this key design element, the introduction of a pathway that allows the uncoupling of growth from heterologous biosynthesis and the establishment of a metabolic valve for the control of pathway fluxes.

<u>Comment 1.2:</u> Enzyme kinetics [K(m) and k(cat)] are of significant importance in the presented (biotechnological) study – values and applied methods have to be included in detail into the main text.

<u>Response:</u> The kinetic parameters [K(m) and k(cat)] of the terpene synthase enzymes and mutants were in the supplementary data section of the submitted manuscript. The corresponding table has now been moved to the main manuscript text as **Table 1** and for some of the enzymes the parameters have also been included in the main text to facilitate the reader **(lines 185-187)**. Furthermore, the methods used for this analysis have been moved from the Supplementary Information to the main materials and methods section **in lines 497-517.**

<u>Comment 1.3:</u> The synthetic metabolism should be confirmed at least in one case by isotope labelling experiments.

<u>Response:</u> We used isotope labelling to add further proof that the synthetic pathway is active and to determine the relative contribution of GPP and NPP in the production of monoterpenes in this system. The findings from these experiments corroborate the results that were already presented in the initial version of the manuscript to establish that the synthetic pathway was functional. Specifically: 1. We had proven production of NPP by converting it to the expected monoterpene products using the NPP-specific tomato enzymes (**lines 109-116; Fig 2A**), which do not convert GPP. The corresponding products were synthesized only when *Sl*NPPS1 was present. 2. We had further confirmed the functionality of the pathway by detecting significant amounts of nerol in the media as a result of NPP overproduction (**lines 118-124; Fig 2B**). Nerol can only be produced by the hydrolysis of NPP, while hydrolysis/dephosphorylation of GPP yields linalool and geraniol. 3. For this reason, nerol was subsequently used in the initial manuscript as a proxy of the NPP pool in the system and its decrease coincided with the efficiency of the production of NPP-derived compounds (**Fig. 2D; Supplementary Table 7; Table 2**).

In the revision, we synthesized ^{13}C-labelled NPP and GPP substrates and used them in feeding experiments to validate that the monoterpenes produced are mainly derived from NPP rather than GPP. In **Supplementary Fig. 10,** we show that when a certain amount of GPP was added to yeast cell

extracts, most of it was converted to FPP and only a small amount could be used to make limonene. By contrast, when NPP was used instead, this was not utilized to make longer prenyl diphosphates but was used exclusively to produce limonene. We continued to show that the limonene produced was almost exclusively derived from ^{13}C-NPP and not from DMAPP- and IPP-derived GPP. **In Supplementary Fig 12**, we provide a competition experiment, where we show that when defined concentrations of ^{13}C-NPP and GPP were added together, limonene was mainly produced by ^{13}C-NPP. At an equal ratio of ^{13}C-NPP:GPP, more than 90% of limonene was produced from ^{13}C-NPP, because GPP was mostly used to make FPP.

Comment 1.4: Figure 4b seems to be incomplete.

Response: Figure 4b has been corrected. Thank you for pointing this out.

Comment 1.5: Even though five different enzymes have been described to accept the alternate substrate, detailed analysis was performed on SfCinS1 – please write a short paragraph why this enzyme was selected.

Response: For the protein engineering studies, we selected to initiate the study with *Sf*CinS1 for several reasons. There was structural information available for this enzyme and we had extensive previous experience in engineering *Sf*CinS1 to change its substrate and product specificity (Kampranis *et al.*, 2007). Furthermore, we had available mutant libraries that facilitated screening to identify residues responsible for isomeric substrate selectivity. This explanation is included in the manuscript in **lines 224-226**.

Comment 1.6: In the abstract, the results and the performed approach appear exaggerated.

Response: The abstract has been adjusted accordingly, and the new results on dynamic regulation have now been included.

Reviewer #2

Comment 2.1: The introduction to the paper is very brief. For example, while the concept of orthogonality has been proposed before, it has not yet prevalent in metabolic engineering. Hence, authors could include more details introducing the concept and how it connects to dynamic regulation (as this is not so obvious at least the way it is written) and how it might different from the orthogonality common in synthetic biology (e.g. noncanonical AAs, orthogonal genetic parts and circuits).

> *Response:* This is very good suggestion. In combination with comment 1.1 of reviewer #1, it is obvious that our explanation of orthogonality was not so clear, and this has led to a misunderstanding of the main findings and their significance. We have now expanded the introduction to explain this better (**lines 41-48;** see also the response to comment 1.1).

Comment 2.2: While the authors have shown that the 5 monoterpene synthases have activity with both NPP and GPP, this reviewer feels it is important to distinguish the extent of production through the orthogonal pathway vs the native pathway for the original as well as the native enzymes.

> *Response:* This is a very good point. However, monoterpene synthases convert both GPP and NPP to the same blend of products (Supplementary Fig. 8), not allowing to determine the relative contribution of the two substrates simply by examining the product profile. To address this comment, we synthesized ^{13}C-labelled substrates (**Supplementary Fig. 9**) so as to distinguish the products of each substrate when a mixture of labelled and unlabeled precursor was used. Using yeast cell extracts incubated with different ^{13}C-NPP: GPP ratios, we were able to determine that, when equal amounts of the two substrates were used, the majority of limonene synthesized by wild-type *Cl*LimS originated from NPP, and that only at 10-times higher GPP concentration the two substrates contributed equally (**Supplementary Fig. 12 left graph**). When the same competition experiment was carried out with the *Cl*LimS(H570Y) mutant, the effect was more pronounced, and over 75% of limonene was produced from NPP at 1:10 NPP:GPP ratio (**Supplementary Fig. 12 right graph**).

Comment 2.3: The manuscript ends a bit abruptly given that the paper is leading towards the dynamic regulation and one would have really liked to see some results on that part but I do understand scope limitations.

> *Response 3:* We felt that this suggestion would greatly improve the manuscript and we decided to implement dynamic regulation. We selected to regulate the flux through Erg20p by introducing an ergosterol-repressive promoter upstream of the *ERG20* gene. This design would help restricting the drain

of precursors when adequate levels of ergosterol are present, shifting fluxes from GPP to NPP synthesis. Indeed, implementing dynamic control resulted in further increases in the titer of limonene and sabinene. The dynamic control design and results have now been included in a dedicated section in the manuscript (**lines 341-354**) and the titers obtained are summarized in **Table 2** and **Fig. 5G**.

Further improvement of the dynamic regulation system by incorporating more elaborate control mechanisms will lead to further titer improvements. This approach expanded the scope of the manuscript and shows how orthogonality considerations and synthetic pathway design can be applied in the engineering of bioproduction systems.

Comment 2.4: For the Figure 4A and the enzyme SfCin did the authors consider mutations at both N338 and F571. These days such mutants can be easily ordered...

Response: We had not initially considered these combinations because the F571 substitutions alone were already quite effective. In the revision, we constructed several of these combinations and tested them, including *Sf*CinS1(N338I-F571H), *Sf*CinS1(N338A-F571H), *Sf*CinS1(N338C-F571H), *Sf*CinS1(I451V-F571H), *Sf*CinS1(N338S-F571H), *Sf*CinS1(N338S-F571V), *Sf*CinS1(N338S-F571L), *Sf*CinS1(N338S-F571I), *Sf*CinS1(N338S-F571Y). The results of this evaluation have been included in an updated version of **Fig. 4A**, and **Supplementary Table 6**. None of the combinations showed an improvement compared to the single mutants (**lines 238-241**) and, therefore, were not pursued further.

Comment 2.5: Figure 1 can be improved to indicate the different products that the authors are considering as well as the terpene synthase enzymes that were being engineered in order to help with the flow of the story.

Response: This was a very good suggestion. We have now updated Figure 1 accordingly.

Reviewer #3

Comment 3.1: "There is some creative metabolic engineering in this work to improve product titers, and there is some thorough protein engineering work to alter the substrate specificity of the monoterpene synthases. However, these results do not support the larger claims of the work around the greater efficacy of an "orthogonal" pathway relative to a native pathway. For example, a central claim of the work is that producing monoterpenes in yeast is difficult because cells are not optimized to produce GPP - whereas producing sesquiterpenes has been successful at scale because yeast cells are optimized for the production of FPP-derived molecules. This doesn't make much sense to me, as cells must produce stoichiometrically equivalent amounts of GPP as FPP, given that GPP is a direct precursor to FPP. Cells are no more optimized to produce FPP than they are optimized to produce GPP. There are far simpler explanations for the observation that monoterpenes have not been as effectively produced in S. cerevisiae - for instance, monoterpenes are generally more toxic to cells than sesquiterpenes."

Response: We agree with reviewer #3 that the reason behind the low monoterpene titers may appear counter-intuitive because the two substrates are connected. However, although the two substrates are stoichiometric, their respective pools are not equal. As shown previously (Ignea *et al.*, 2014), the GPP pool is much smaller than the FPP pool because of the sequential mechanism of Erg20p. This sequential mechanism is advantageous for yeast cells because they do not use GPP to synthesize other compounds than FPP. But it is a hinderance for the production of monoterpenes because Erg20p removes GPP very efficiently. However, since Erg20p and sterols are essential, an alternative approach is needed. Introducing an orthogonal substrate for monoterpene synthesis (NPP), uncouples Erg20p and sterol biosynthesis from monoterpene production, allowing to divert fluxes to the synthetic branch.

To corroborate the earlier findings and provide further evidence to address the reviewer's concerns, we carried out additional experiments to demonstrate: 1. the efficient conversion of GPP to FPP that drains the GPP pool, and 2. that toxicity is not a limiting factor under these conditions.

1. We fed pathway precursors (DMAPP and IPP) to extracts of yeast cells expressing limonene synthase (*Cl*LimS) and determined the amounts of GPP and FPP produced by the precursors and limonene produced by GPP. We confirmed that DMAPP and IPP were efficiently utilized to produce FPP, but only limited amounts of GPP and GPP-derived limonene were determined. Supplementing the precursors with GPP again resulted mainly in the production of FPP and only small amounts of limonene. By contrast, when NPP was used instead of GPP to supplement this system, this was readily converted to limonene and not taken further to other prenyl diphosphates. Using ^{13}C-NPP, we confirmed that the limonene produced in this experiment originated almost entirely from the labelled substrate. Considering that *Cl*LimS prefers GPP to NPP (**Table 1**), these results confirm

that utilizing an orthogonal substrate is advantageous for monoterpene synthesis. These experiments are now presented in **Supplementary figures 1, 10 and 11** and are discussed in **lines 75-77 and 199-209**.

2. Furthermore, we carried out experiments to show that toxicity was not the reason for the previously observed low productivity. First, we showed that supplementing yeast cultures with concentrations of monoterpenes that exceeded by 10 times the previous maximum reported titer did not inhibit the growth of yeast cells (**Supplementary Figure 2**). Subsequently, we confirmed that the productivity of limonene-producing yeast cells was not reduced, even when the culture was supplemented with 10-fold higher exogenous limonene (**Supplementary Fig. 3**). Taken together, these results confirm that cell growth and productivity are not inhibited by the monoterpene product in this titer range.

Comment 3.2: The authors also claim that production of monoterpenes in E. coli is higher than in S. cerevisiae because there are no major biomass generating pathways that depend on GPP. This is an important prediction of their "orthogonality" claim, but there is no actual evidence that the lack of competing pathways in E. coli has anything to do with the higher titers. In fact, it would logically follow from this claim that production of sesquiterpenes should also be more efficient in E. coli, which is clearly not the case.

Response: Sesquiterpene production in *E. coli* is very efficient. For example, titers of 27 g/L amorpha-4,11-diene were achieved quite early in metabolic engineering efforts (Tsuruta *et al.*, 2009). Monoterpene titers in *E. coli* are quite lower than sesquiterpene titers, currently in the 0.5-1.5 g/L range, e.g. (Mendez-Perez *et al.*, 2017). Thus, in *E. coli*, sesquiterpene production compares favorably to monoterpene production. Although there must be several additional factors that contribute to the observed titers, their relative comparison is in agreement with the orthogonality hypothesis,

The challenge with terpenoid production in *E. coli,* and the reason that yeast has been the preferred host for the production of many compounds, is mostly the inability to perform efficient cytochrome P450-mediated oxidations using membrane-bound enzymes in *E. coli* (Paddon & Keasling, 2014). Thus, establishing efficient production of monoterpenes in yeast will enable us to move beyond the synthesis of hydrocarbons to the production of oxidized industrially relevant molecules (e.g. menthol). This was explained in the discussion (**lines 368-371**).

Comment 3.3: The idea that the NPP synthase offers more "orthogonality" than simply introducing a monoterpene synthase is also a subjective one. It could just as easily be claimed that the NPP synthase-using pathway is a two-step heterologous pathway to produce monoterpenes drawing from IPP/DMAPP, whereas the "canonical" method to product monoterpenes is a one-step pathway drawing from GPP. The idea of "orthogonality" seems semantic, rather than directly relevant to their results. The higher levels of production in

the presence of the NPP synthases could be explained by enzyme kinetics as much as pathway structure - this simpler explanation needs to be ruled out before claims of orthogonality could be made.

> *Response:* 1. To confirm that the effect was not due to the kinetic characteristics of *Sl*NPPS1, we produced the recombinant protein (**Supplementary Figure 4B**) and determined its kinetics properties. We found that *Sl*NPPS1 is significantly less efficient than Erg20p (**Supplementary Table 4**). Similarly, most wild-type canonical terpene synthases used show favorable kinetics with GPP than NPP (**Table 1**). Thus, the observed titer improvements cannot be explained by the kinetic characteristics of the enzymes involved in the NPP branch.

Comment 3.4: There are also some very basic experimental issues with the paper. For example, in many of the figures, "samples were analyzed in triplicates." To me this sounds like technical replicates - am I misunderstanding this? Biological replicates are necessary to understand the reproducibility of these results - technical replicates are not the the appropriate test for establishing reproducibility. The language makes me think that the authors performed only technical replicates for many of the experiments.

> *Response:* There are both biological and technical replicates, depending on the experiment. All main findings are from biological replicates from the point of yeast transformation. We have included a clear explanation in each figure about what the replicates correspond to. We are also providing an extensive **Source Data file**, where all the raw data from our experiments are shown, figure-by-figure. Furthermore, we have adjusted all figures to include the **actual data points** in addition to the average and errors. We are confident that the provided information will be helpful to the reader to validate the reproducibility of the results.

Comment 3.5: I am also very puzzled by the lack of information around the last claim of the work, which is that the NPP-specific synthases enabled production of monoterpenes with higher efficiency than the native pathway. Given that record monoterpene titers are a primary claim in the abstract, these results need to be in a figure, properly described, with described methods, appropriate statistical analysis and genotypic description of strains. I could not tell from the manuscript how the authors achieved these titers of 130 mg/L and 72.7 mg/L, and I do not see any genotype information for these strains. The description of their control, "the same strain by the native pathway," is very vague. What is meant by the "native pathway" here? Does it include any of the Erg20 engineering shown in Figure 2? None of this information is provided. Moreover, it seems like the logical experiment would be to put the newly engineered NPP-specific synthases into the strains constructed for Figure 2 and determine whether there is a large increase in monoterpene levels, and a decrease in NPP levels.

Response: We agree that the specific part was not described in sufficient detail. Indeed, the experiment performed in this paragraph was the experiment recommended by the reviewer, where the engineered synthases were introduced in the optimized yeast strain. We have rewritten this whole section to clarify the experiments (**lines 320-324**). Because in the revised version we have expanded the experimental part by introducing dynamic regulation, we have now added a whole new paragraph in **lines 341-354** and provide additional tables where the strains and yields are clearly described (**Table 2; Supplementary Table 7, Source Data**). We have also included an additional figure (**Fig. 5G**) that demonstrates the dynamic valve introduced and the titers obtained. Moreover, we have extended the use of nerol as a proxy for excess NPP and show how this decreases as product titers increase (**Supplementary Table 7; Table 2**). Strain genotypes are provided in **Supplementary Table 1**.

We feel that the clarification of the approach together with the additional experimental evidence presented in the revised version convincingly support the main claims of the manuscript and make the novelty of the findings more evident.

References:

1 Dunlop, M. J. *et al.* Engineering microbial biofuel tolerance and export using efflux pumps. *Mol. Syst. Biol.* **7**, 487 (2011).

2 George, K. W. *et al.* Integrated analysis of isopentenyl pyrophosphate (IPP) toxicity in isoprenoid-producing Escherichia coli. *Metabol. Eng.* **47**, 60-72 (2018).

3 Todorovic, B. *et al.* Toxicity of twenty-two plant essential oils against pathogenic bacteria of vegetables and mushrooms. *J. Environ. Sci. Health. B.* **51**, 832-839 (2016).

4 Jongedijk, E. *et al.* Biotechnological production of limonene in microorganisms. *Appl Microbiol Biotechnol.* **100**, 2927-2938 (2016).

5 Denby, C. M. *et al.* Industrial brewing yeast engineered for the production of primary flavor determinants in hopped beer. *Nat. Commun.* **9**, 965 (2018).

6 Brennan, T. C., Turner, C. D., Kromer, J. O. & Nielsen, L. K. Alleviating monoterpene toxicity using a two-phase extractive fermentation for the bioproduction of jet fuel mixtures in Saccharomyces cerevisiae. *Biotechnol. Bioeng.* **109**, 2513-2522 (2012).

7 Pandit, A. V., Srinivasan, S. & Mahadevan, R. Redesigning metabolism based on orthogonality principles. *Nat. Commun.* **8**, 15188 (2017).

8 de Lorenzo, V. Beware of metaphors: chasses and orthogonality in synthetic biology. *Bioeng. Bugs* **2**, 3-7 (2011).

9 Mendez-Perez, D. *et al.* Production of jet fuel precursor monoterpenoids from engineered Escherichia coli. *Biotechnol Bioeng* **114**, 1703-1712 (2017).

10 Ignea, C., Pontini, M., Maffei, M. E., Makris, A. M. & Kampranis, S. C. Engineering monoterpene production in yeast using a synthetic dominant negative geranyl diphosphate synthase. *ACS Synth. Biol.* **3**, 298-306 (2014).

11 Tsuruta, H. *et al.* High-level production of amorpha-4,11-diene, a precursor of the antimalarial agent artemisinin, in Escherichia coli. *PloS One* **4**, e4489 (2009).

12 Paddon, C. J. & Keasling, J. D. Semi-synthetic artemisinin: a model for the use of synthetic biology in pharmaceutical development. *Nat. Rev. Microbiol.* **12**, 355-367, (2014).

Reviewers' comments:

Reviewer #1 (Remarks to the Author):

In the revised version Ignea et al. carefully addressed all points raised. The re-submitted version is excellent and the added experiments are of high quality. I recommend the acceptance of the mauscript and have no suggestions for additional changes.

Reviewer #2 (Remarks to the Author):

Authors have made most of the changes requested in the previous version. Specifically they add an experiment around the dynamic regulation of the ERG20 pathway that acts as a metabolic valve. While this is valuable, it opens a few more questions. One is what is the rationale for the choice of the valve, is it possible to provide a justification for this. There have been recent computational methods for the identification of valves for dynamic control (Venayak, von Kamp, Mahadevan, 2018). In addition, the details of how the valve was triggered is missing. For example, authors claim that it was ergosterol sensitive switch and it seems as if they added terbinafine or ketonazol but it is not clear and the methods are not detailed. Also, it is not clear how much of an impact the timing of this switch. This aspect is not discussed. Perhaps authors can incorporate these changes in their revised paper.

Reviewer #3 (Remarks to the Author):

I have two main comments. One is about the work, and one is about the presentation of the state of the field.

Regarding the work: I believe the work is improved by the detailed experiments added, and the authors have ruled out some obvious alternative hypotheses to why their pathway is more efficient. There are some well-done and effective applications of metabolic engineering and enzyme engineering approaches in this body of work, leading to some nice results.

Regarding the presentation of the state of the field: This manuscript would be much improved if there were more caution in the assessment and interpretation of the literature. For instance, from the introduction: "However, production of GPP derived compounds has so far been considerably less efficient. This can be attributed to the structure of the isoprenoid biosynthesis pathway in yeast, which is optimized for the production of FPP derived molecules that are essential for growth and viability." And, from the conclusion, "So far, efforts in monoterpene bioproduction have been more efficient when using bacterial hosts because in these cells there are no major biomass generating pathways that depend on GPP." The authors are asserting that the varying degrees of success in monoterpene production are related to the structure of the metabolic network. However, this is conjecture.

Yes, the authors demonstrate that they can boost monoterpene production through introduction of their pathway. However, it does not logically follow that lack of an orthogonal pathway must be what is preventing economical monoterpene production by the field in general.

Subject: Response to reviewer comments (NCOMMS-18-29430A)

We would like to sincerely thank the reviewers for their positive evaluation of the revised manuscript and for their constructive additional comments. We have addressed all their points by providing additional information or justification and revising sections of the manuscript accordingly. Please find below our point-by-point response:

Reviewer #1: In the revised version Ignea et al. carefully addressed all points raised. The re-submitted version is excellent and the added experiments are of high quality. I recommend the acceptance of the manuscript and have no suggestions for additional changes.

> *Response:* We would like to thank reviewer #1 for the useful comments during the revision process and for recommending acceptance of the revised manuscript.

Reviewer #2: Authors have made most of the changes requested in the previous version. Specifically they add an experiment around the dynamic regulation of the ERG20 pathway that acts as a metabolic valve. While this is valuable, it opens a few more questions.

Comment 2.1: One is what is the rationale for the choice of the valve, is it possible to provide a justification for this. There have been recent computational methods for the identification of valves for dynamic control (Venayak, von Kamp, Mahadevan, 2018).

> *Response:* The position of the valve was selected by inspecting the structure of the constructed pathway in relation to the endogenous sterol pathway (Fig. 1). The Erg20p-catalyzed step seemed to be the obvious intervention point as it is the first step in the sterol branch. For the regulation of the valve, we selected the ergosterol-sensitive P_{ERG1} promoter because this system had previously been successfully used for the downregulation of sterol synthesis in two occasions. In the production of amorphadiene by controlling the *ERG9* gene and in the production of casbene by controlling both the *ERG9* and *ERG20* genes. (Yuan & Ching, 2015; Callari et al., 2018). We considered that this approach would also be appropriate to establish proof-of-concept for the positive effect of dynamic control in our system. This information has now been included in **lines 321-327** of the revised manuscript.
>
> For further improvement of the current set up, more advanced methods for the identification of valves for dynamic control, like the metabolic valve enumerator (MoVE) computational method (Venayak et al., 2018), can be used. This is discussed briefly in the discussion section (**lines 343-345**).

Comment 2.2: In addition, the details of how the valve was triggered is missing. For example, authors claim that

it was ergosterol sensitive switch and it seems as if they added terbinafine or ketonazol but it is not clear and the methods are not detailed. Also, it is not clear how much of an impact the timing of this switch. This aspect is not discussed. Perhaps authors can incorporate these changes in their revised paper.

Response: The valve is regulated by ergosterol, which controls a negative regulatory DNA element in P_{ERG1}. The addition of terbinafine or ketonazol was carried out only in the initial experiments that characterized the behavior of P_{ERG1} by (Leber et al., 2001). The effect of the inhibitors was mentioned only to explain the rationale behind using this specific control mechanism and was removed in the revised version to avoid confusion. Although we did not study in detail the behavior of this switch in our system, based on the observations of previous studies where it has been applied (Callari et al., 2018), we consider that this regulation is likely continuous during growth and beneficial for the heterologous production throughout the growth phase. To better explain the design and function of this valve, we rephrased parts of the dynamic regulation section to clarify this (**lines 321-327**).

Reviewer #3: I have two main comments. One is about the work, and one is about the presentation of the state of the field. Regarding the work: I believe the work is improved by the detailed experiments added, and the authors have ruled out some obvious alternative hypotheses to why their pathway is more efficient. There are some well-done and effective applications of metabolic engineering and enzyme engineering approaches in this body of work, leading to some nice results.

Comment 3.1: Regarding the presentation of the state of the field: This manuscript would be much improved if there were more caution in the assessment and interpretation of the literature. For instance, from the introduction: "However, production of GPP derived compounds has so far been considerably less efficient. This can be attributed to the structure of the isoprenoid biosynthesis pathway in yeast, which is optimized for the production of FPP derived molecules that are essential for growth and viability." And, from the conclusion, "So far, efforts in monoterpene bioproduction have been more efficient when using bacterial hosts because in these cells there are no major biomass generating pathways that depend on GPP." The authors are asserting that the varying degrees of success in monoterpene production are related to the structure of the metabolic network. However, this is conjecture. Yes, the authors demonstrate that they can boost monoterpene production through introduction of their pathway. However, it does not logically follow that lack of an orthogonal pathway must be what is preventing economical monoterpene production by the field in general.

Response: Overall, we would like to thank reviewer #3 for the constructive comments in the previous revision that helped improve the manuscript. We understand the concerns of reviewer #3 regarding the clear

description of the contribution of this work and we reviewed the manuscript text to edit phrases or expressions that could lead to misinterpretations. To this end:

1. We have rephrased the two sentences highlighted here by the reviewer in the introduction **lines 64-65**) and discussion (**lines 348-349**) to explain that the structure of the metabolic network is only one of the limiting factors.
2. We have also carefully reviewed the whole manuscript and rephrased a few more sentences to make the outcomes of this work clearer and to avoid any phrasing that may imply that introduction of this pathway is by itself sufficient to achieve economical monoterpene production (**line 50, lines 71-72, line 81**).

REFERENCES

1 Yuan, J. & Ching, C. B. Dynamic control of ERG9 expression for improved amorpha-4,11-diene production in Saccharomyces cerevisiae. *Microb Cell Fact* **14**, 38 (2015).

2 Callari, R., Meier, Y., Ravasio, D. & Heider, H. Dynamic Control of ERG20 and ERG9 Expression for Improved Casbene Production in Saccharomyces cerevisiae. *Front. bioeng. biotechnol.* **6**, 160 (2018).

3 Venayak, N., von Kamp, A., Klamt, S. & Mahadevan, R. MoVE identifies metabolic valves to switch between phenotypic states. *Nat Commun* **9**, 5332 (2018).

4 Leber, R. *et al.* A novel sequence element is involved in the transcriptional regulation of expression of the ERG1 (squalene epoxidase) gene in Saccharomyces cerevisiae. *Eur. J. Biochem.* **268**, 914-924 (2001).

REVIEWERS' COMMENTS:

Reviewer #1: Unavailable.

Reviewer #2 (Remarks to the Author):

Thank you for editing the paper to include the changes. Congratulations on this nice work !!

Reviewer #3 (Remarks to the Author):

I'm glad to see that the authors found a way to explain the concepts and thesis behind network structure optimization and how it can be used to improve monoterpene production, while successfully avoiding excessive generalization about the field in general. I believe these changes give the manuscript more credibility, and will help readers focus their attention on the very interesting experiments and results.
